# Using collective intelligence methods to improve government data infrastructures and promote the use of complex data: The example of the Northern Ireland Longitudinal Study

**DOI:** 10.1186/s12961-023-01070-x

**Published:** 2023-12-18

**Authors:** Estelle Lowry, Michael J. Hogan, John Moriarty, Owen M. Harney, Erna Ruijer, Monika Pilch, Jenny M. Groarke, Michelle Hanlon, Ian Shuttleworth

**Affiliations:** 1 School of Natural and Built Environment, Queen’s University Belfast, University Street, BT7 1NN Belfast, Northern Ireland; 2 School of Psychology, University of Galway, Galway, Ireland; 3 School of Social Sciences, Education and Social Work, Queen’s University Belfast, BT7 1NN Belfast, Northern Ireland; 4 School of Medicine, Trinity College Dublin, Dublin, Ireland

**Keywords:** Data, Open access, Data infrastructure, Collective intelligence

## Abstract

**Background:**

This paper discusses how collective intelligence (CI) methods can be implemented to improve government data infrastructures, not only to support understanding and primary use of complex national data but also to increase the dissemination and secondary impact of research based on these data. The case study uses the Northern Ireland Longitudinal Study (NILS), a member of the UK family of census/administrative data longitudinal studies (UKLS).

**Methods:**

A stakeholder-engaged CI approach was applied to inform the transformation of the NILS Research Support Unit (RSU) infrastructure to support researchers in their use of government data, including collaborative decision-making and better dissemination of research outputs.

**Results:**

We provide an overview of NILS RSU infrastructure design changes that have been implemented to date, focusing on a website redesign to meet user information requirements and the formation of better working partnerships between data users and providers within the Northern Ireland data landscape. We also discuss the key challenges faced by the design team during this project of transformation.

**Conclusion:**

Our primary objective to improve government data infrastructure and to increase dissemination and the impact of research based on data was a complex and multifaceted challenge due to the number of stakeholders involved and their often conflicting perspectives. Results from this CI approach have been pivotal in highlighting how NILS RSU can work collaboratively with users to maximize the potential of this data, in terms of forming multidisciplinary networks to ensure the research is utilized in policy and in the literature and providing academic support and resources to attract new researchers.

**Supplementary Information:**

The online version contains supplementary material available at 10.1186/s12961-023-01070-x.

## Background

Governments all around the world are experimenting with collaborative data infrastructures in an effort to support deliberative and participatory approaches to local and national policy development and project implementation. Data can foster collaboration, promote greater openness and create real-time solutions to public challenges such as health, education, transportation and support policy and decision-making [1]. Data may consist of longitudinal survey studies, publicly archived research data, linked administrative data and open government data [2, 3]. Ongoing infrastructure design projects focus on key issues of data linkage, and affordances to make data accessible, understandable and usable [4]. Comprehensive national data infrastructures provide particular advantages associated with the linkage of population-level administrative records to create individual-level microdata which is important in relation to addressing societal challenges. Particularly, in the context of formulating and refining national health policies, operating at a population scale improves both statistical power and representativeness, while also allowing researchers to detect health risks to small sub-groups, often not adequately captured in sample-based research [5]. Crucial also is the ability to thoroughly contextualize health events through information from other sources on socioeconomic conditions and features of the lived environment. Contemporaneous records, for example, of employment or housing tenure status, or of health events, eliminate issues of recall and other errors characteristic of survey research [6].

Although researchers, citizens and public administrators are increasingly making use of data, data usage is still in its infancy [7–9]. Furthermore, many data infrastructures do not stimulate or support data use [9]. Consequently, in efforts to inform better data infrastructure design, scholars have sought to understand what determines open data usage [7, 8, 10].

Several scholars take a reductionist user-oriented approach and focus on identifying user and innovation barriers that impact data usage [11]. For example, there are technical barriers (including a lack of data, poor data quality and lack of sufficient metadata), innovation barriers (for example, lack of transparency, lack of focus on user needs and reluctance of government bodies) and social barriers (for example, lack of communication, lack of knowledge and skills, and lack of awareness of data and its knowledge potential) [11–17]. While it is important to identify specific user-oriented barriers and needs, data infrastructure design also implies a systems thinking perspective, including reflection on how a range of needs are facilitated in the overall system design.

Aligned with the systems thinking perspective, other scholars take a holistic approach and analyse data usage within an ecosystem [18–21]. Data ecosystems are composed of public, private and non-profit actors playing specific roles related to the usage of data, and of specific functions such as data gathering, data provision, data usage and an intermediation function between providers and users [19]. Data ecosystem approaches focus not only on the different components of data programs but also on their dynamic relationships and the way in which different components and relationships influence data usage [18]. To stimulate data usage, the actors need to create a mutual relationship of trust, allowing data to flow from the public sector to communities of users via intermediaries and back to policy-makers [19]. Communication and feedback mechanisms are components of this approach. However, to date, few studies have focused on communication and dissemination processes supporting awareness and use of data. Chokki et al. identify different communication methods such as the use of social media, public outreach campaigns, workshops, training, hackathons and applications. The latter focus on applications includes infrastructures to help users easily access data and tangible examples of what can be done with data [12].

Complex design challenges are increasingly being addressed through the application of collective intelligence (CI) methodologies [22–25]. CI refers to the combined capacity of a group to solve shared problems. In this study, a key starting point for CI design is a focus on existing applications, communication methods and dissemination strategies that are part of the Northern Ireland Longitudinal Study (NILS) Research Support Unit (RSU) infrastructure, which in turn provides a catalyst for envisioning new infrastructure design affordances. We contribute to the literature on data usage by linking reductionist and holistic approaches, using iterative design and transformation of user support infrastructures. Stakeholder-engaged CI design does not only focus on identifying specific user-oriented barriers and needs but also on barriers and needs linked to inter-institutional coordination and intermediation functions that influence how accessible, understandable and usable data is to users.

### The Northern Ireland Longitudinal Study

In the early 2000s, a group of academics and senior statisticians from the Northern Ireland Statistics and Research Agency (NISRA) was convened to explore the required costs and infrastructure needed to form a Northern Ireland Longitudinal Study [26]. This would be similar in design to longitudinal studies already operating in England and Wales, and under development in Scotland. The aim was to have a multi-cohort study that would fulfil a range of academic and policy-related purposes, with a sample size large enough to enable robust analysis of population subgroups and of areas of policy relevance.

Following the launch of NILS and the Northern Ireland Mortality Study (NIMS) in 2006, the funding model has evolved slightly over the years, and all funding for development and maintenance of the NILS is via the Health and Social Care Research and Development Division of the Public Health Agency and Economic Social and Research Council (ESRC). Additionally, NISRA helps to fund the NILS/NIMS project both through the provision of accommodation to house all aspects of the NILS/NIMS operation and staff to maintain and develop the databases.

**Fig. 1:**
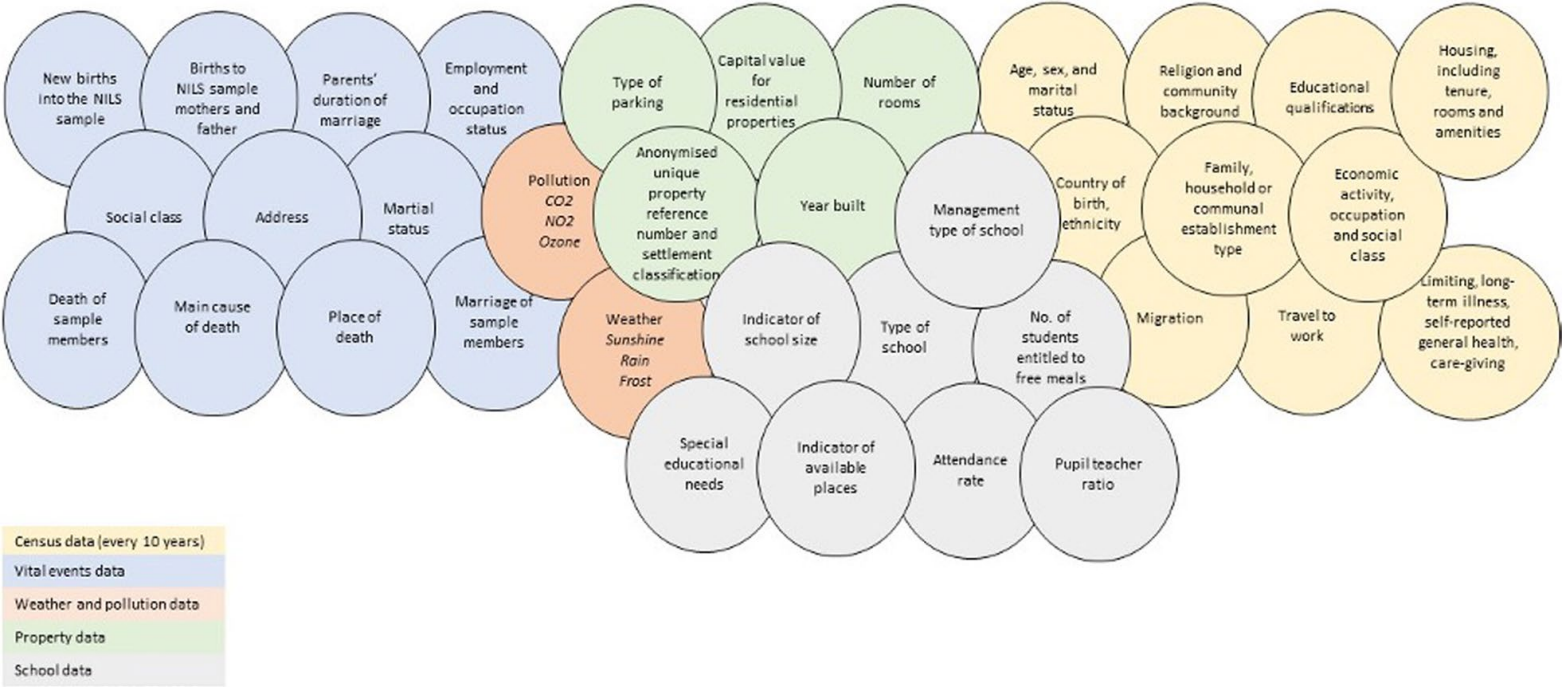
Field representation of core NILS variables

Due to the rich data contained within the NILS and the large, representative sample, it offers an opportunity to address a broad range of research questions. The NILS provides a mechanism for understanding the population health dynamics of Northern Ireland by reference to a range of demographic, health and socio-economic characteristics. An overview of the NILS data variables is presented in Fig. 1, and a more detailed list can be found from the website (www.nils.ac.uk). Diverging from the other longitudinal studies, the NILS uses a health-card spine to which census data are linked. The health-card spine is linked to the 1991, 2001 and 2011 Censuses (with a partial link to 1981) and will soon add the 2021 Census. As the NILS and linked census data use unique health and social care numbers, the benefits are regular biannual updates on vital events and address changes and the possibility for additional distinct linkage projects (DLPs), linking health system data. For example, a study by Ross et al. on breast screening attendance used the NILS linked to breast screening records which revealed that attendance was lower in those women with chronic disabilities and self-reported poor mental health. This highlights the need to re-evaluate the system to find ways to increase attendance in these vulnerable groups of women [27].

**Fig. 2:**
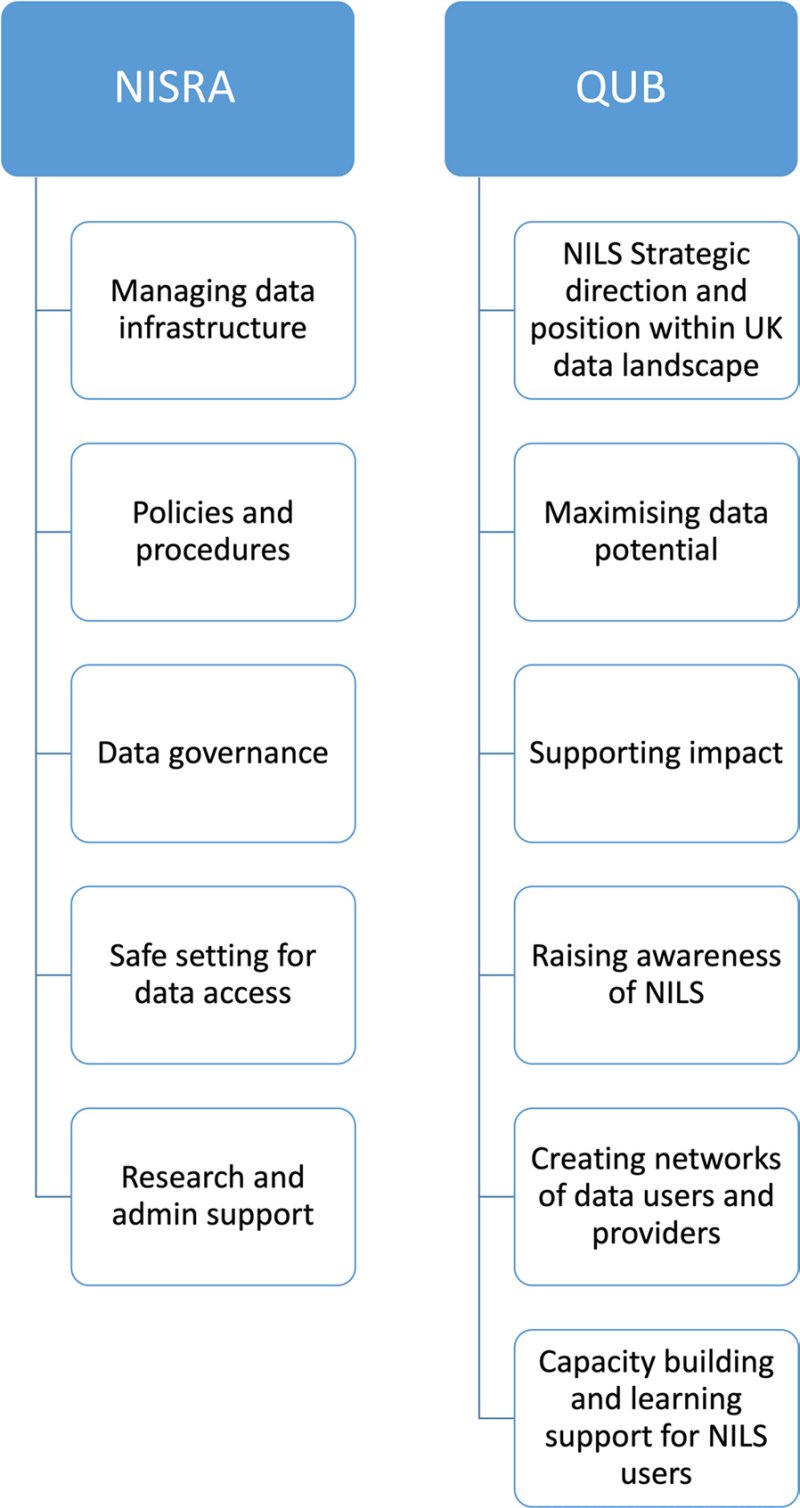
Overview of functions provided by NILS Research Support Unit

Since 2011, the ESRC have typically awarded NILS funding in 5-year cycles with some shorter interim grants, as in 2017. The focus over these funding periods has shifted from Census 2011 linkage to increasing the usage of NILS by expanding the available routine data and widening the user base beyond academia. Primary functions of the RSU are divided among NISRA and Queen’s University Belfast staff on the basis of skill set (Fig. 2). These consist of managing the data infrastructure, policies and procedures; accommodating a safe setting to access the data; providing researcher and administrative support; ensuring the research potential of the data is maximized and impact is achieved by creating networks and partnerships of data users and providers; building capacity; and providing learning support for new and existing researchers.

Using the NILS as a case study, we aim to demonstrate how the implementation of a collective intelligence approach can improve the data infrastructure. In this instance, we are defining the data infrastructure as the basic systems and services required to allow the data to be maintained and used effectively. This encompasses both maintenance of the integrity and quality of the dataset itself but also support for new and existing researchers, which may be in the form of co-designing their research question, capacity-building and/or achieving impact through dissemination. The NILS contains extremely sensitive data, and great care is taken to ensure appropriate and safe use. They are managed by NISRA under census legislation, and access is strictly controlled and governed by protocols and procedures that ensure data confidentiality. The full ethical and legal considerations are detailed on the NILS website and profile paper [26].

## Methods

In this study, the NILS RSU have adopted a scenario-based CI design methodology which builds upon the approach developed by Ruijer et al. In the Netherlands, Ruijer et al. demonstrated how Groningen’s regional open-data repository could be applied to help address the particular policy challenge of population decline. To facilitate infrastructure design focused on how open government data could be accessed, understood and used to address this policy challenge, the design team worked with public administrators, researchers, data experts and technology design experts using an integrated CI methodology [4] comprising interactive management (IM) methodology [28], scenario-based design (SBD) [29] and agile user story [30] methods. The IM method is used to address complex problems and typically involves idea generation, field representation and systems thinking tools which allow groups to think clearly and arrive at a consensus in relation to the nature of a complex problem and solutions [23]. SBD methodologies use scenarios as stories about people and their activities which allow multiple views of interaction and evoke reflection on a range of concrete and specific user needs [29]. Finally, these needs were written in accordance with agile user story idea-writing methods. This allowed singular needs and reasons for needs to be specified in a way that supports higher-level analysis and synthesis of needs across multiple participants in a CI session [4 p. 472]. Utilizing this approach allowed the RSU design team to:

- Identify challenges in accessing, understanding and using NILS data for research purposes and to guide policy, practice and action.
- Clarify specific issues associated with current RSU resources, including the NILS data dictionary, NILS website and NILS data request form.
- Generate user needs to support NILS infrastructure redesign, in particular, information needs, collaboration and decision-making needs and training needs of NILS users.

**Fig. 3:**
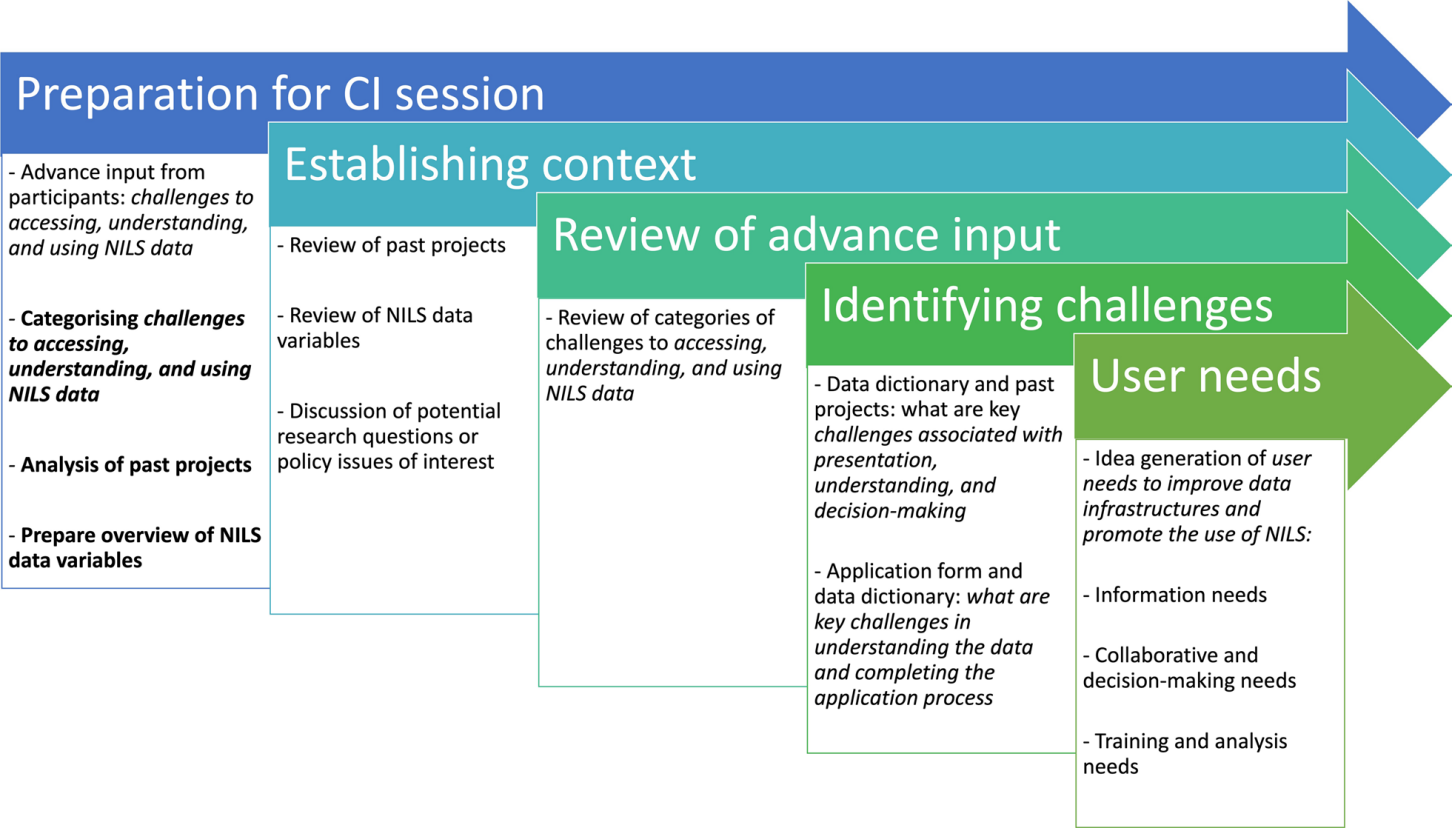
Overview of the CI process. Bold font denotes tasks completed by the facilitation team

**Fig. 4:**
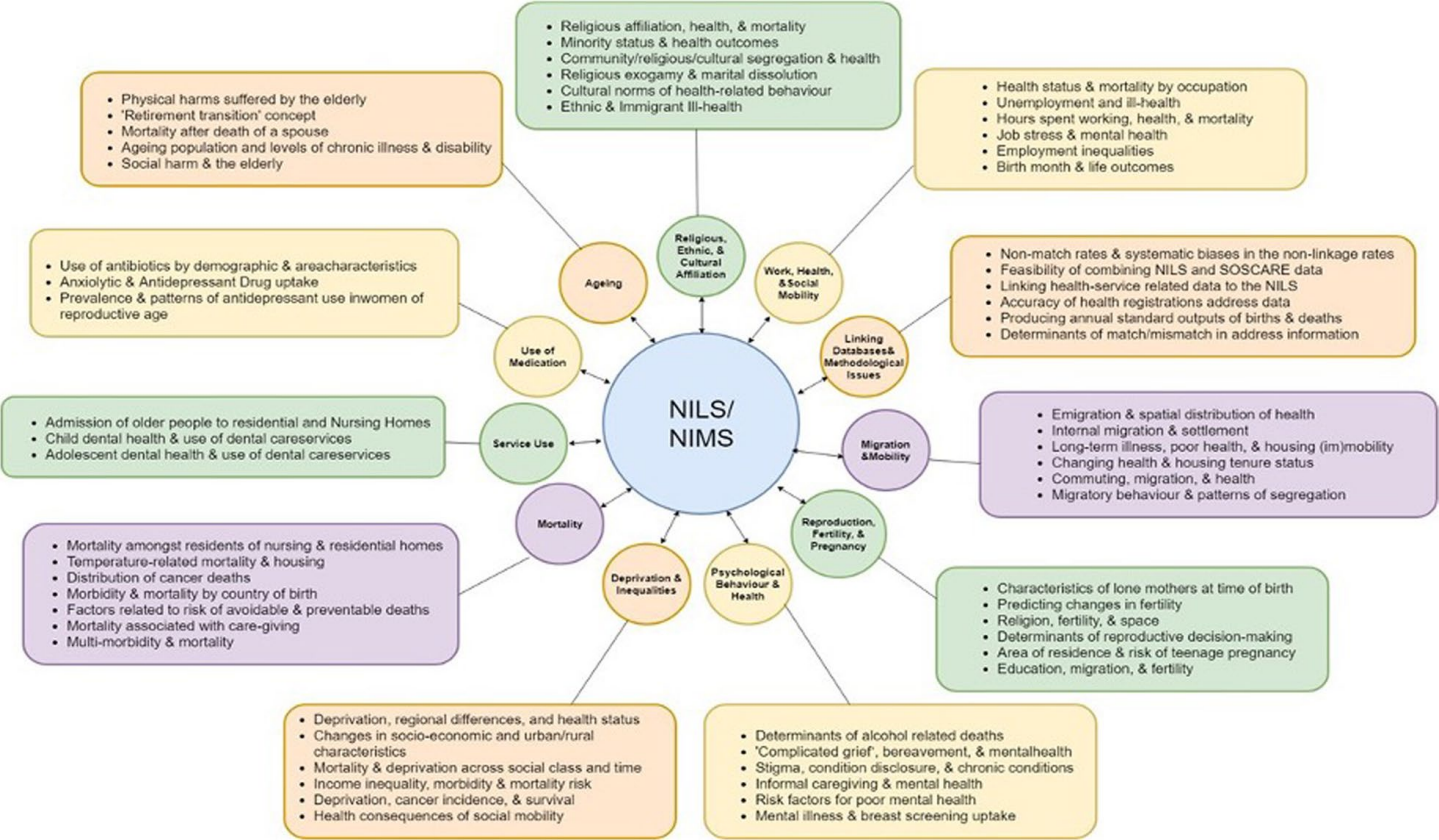
A field representation of NILS projects

An overview of the CI process is summarized in Fig. 3 (see Additional file 1: Appendix A for further information on the methodology). In advance of engaging stakeholders during the face-to-face CI workshop to focus on NILS RSU infrastructure design, the CI facilitation team conducted an analysis of all past NILS projects to identify key themes and issues addressed in previous projects (Fig. 4).

### Collective intelligence session stakeholders

Stakeholders included five government statisticians representing data providers/custodians, eight researchers and five officials with an impact and policy interest who could provide insight into dissemination. Furthermore, there was attendance from the census office to also provide representation of public interest. These stakeholders were selected because they all had experience with the NILS and related longitudinal studies, or represented groups who work with NILS data. All workshop participants agreed to participate in the workshop and collective intelligence design process via email and as part of their commitment to supporting NILS infrastructure redesign.

### Establishing context

An overview of the NILS data variables, along with the categorized field representation of past projects (Figs. 1 and 4), was presented to 18 stakeholders at the CI session for review. After this initial presentation, CI workshop participants were prompted to identify key research or policy issues that they would like to address using NILS data. In particular, participants were asked to identify key variables of interest using the data field representation in Fig. 1, and draft and share ideas across four subgroups. Participants were divided across these subgroups such that there was a balance of gender and diversity of NILS stakeholder experience across groups. A broad array of potential research question ideas were discussed including, for example, whether being an artist influenced longitudinal health outcomes relative to other occupations, and the relationship between air pollution levels and school attendance (that is, absence due to illness) across districts. These ideas were not recorded; rather, this dialogue provided an opportunity for establishing context for the core CI session tasks. This discussion served to prompt consideration of the opportunities and potential offered by NILS data, and to prime stakeholders for further idea generation at the next stage.

### Challenges to accessing, understanding and using NILS data

Participants were next prompted to focus on key design challenges for the NILS RSU team. In advance of the CI session, participants were each invited via email to submit five challenges in response to the following trigger question:

*“What are key challenges to accessing, understanding, and using NILS data for research purposes and to guide policy, practice, and action?”*

This advance input was compiled by the facilitation team, and presented in the form of a printed handout for review by participants during the CI session. This review was followed by a second round of idea generation, which was carried out across the four subgroups during the CI session. This second round focused on specific components of the NILS application process, with each question being addressed by two groups:

*“Upon reviewing the data dictionary and past projects section of the website, what are key challenges associated with presentation, understanding, and decision-making?”*

and.

*“Upon reviewing the NILS application form and data dictionary, what are key challenges in understanding the data and completing the application process?”*

### Mapping user needs

Workshop participants next engaged in a series of SBD exercises focused on generating a comprehensive set of NILS user needs. A set of narrative scenarios describing potential interactions between NILS stakeholders were developed and used to prompt design thinking in relation to:

- Information needs
- Collaborative and decision-making needs
- Training and analysis needs.

Information needs refer to information designs supporting discovery and understanding, relevant data and past findings, and tools supporting navigation of relevant information.

Collaborative and decision-making needs involve consideration of the types of collaboration and decision-making tools, methods and communication processes that are needed.

Training and analysis needs refer to the types of advice, supports, training programme content, procedures and methods that are needed to support analysis and other relevant interactions with NILS data.

This scenario-based design method was combined with ideawriting to maximize the needs developed by workshop participants during the allotted time.

For the purposes of this task, the ideawriting sheets presented multiple agile user story prompts for answering the stimulus question, each presented in the following format:

As User Type _______, I want ______, so that I can ______.

**Fig. 5:**
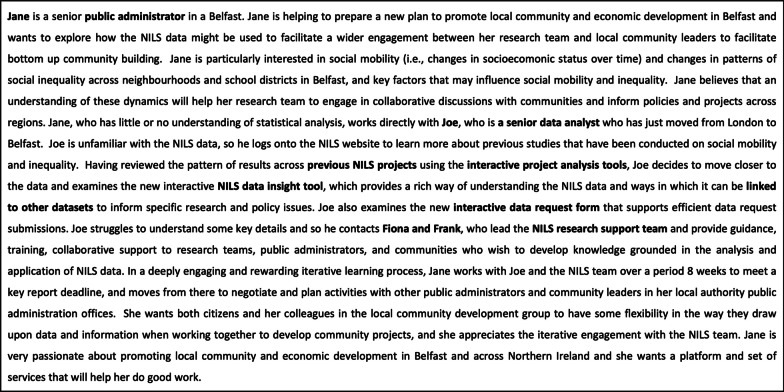
Example scenario from the ideawriting sheets presenting multiple agile user story prompts

One of the scenarios is presented in Fig. 5. The second scenario is presented in Additional file 2: Appendix B.

### Analytical approach

Analysis of textual data generated by participants in advance of and during the CI session, involved the application of a descriptive and exploratory approach, informed by qualitative content analysis [31, 32] (Elo and Kyngäs, Elo et al.). Qualitative analysis for the purposes of identifying categories of challenges was done manually (that is, it was not computer-based) using the paired comparison method [33] (Warfield and Cárdenas). After being immersed in the data, the researchers engaged in a process of systematically assessing challenges for conceptual similarity. Pairs of challenges were considered in turn, in an exhaustive and immersive process. This process resulted in the emergence of higher-order categories of conceptually similar challenges.

## Results

### Challenges to accessing, understanding and using NILS data for research purposes

**Fig. 6:**
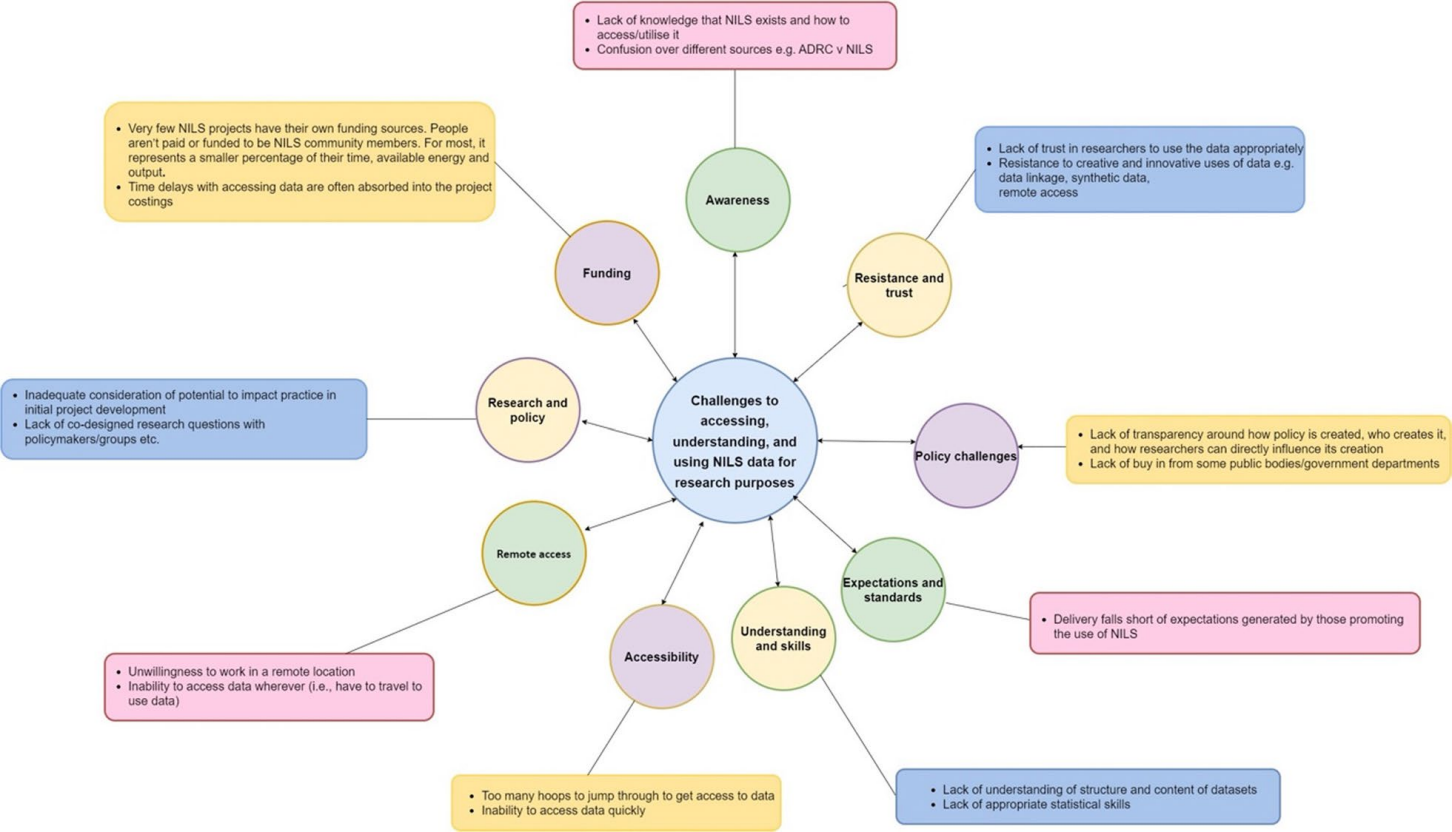
Summary of challenges to accessing, understanding and using NILS data for research purposes

A total of 30 challenges to accessing, understanding and using NILS data for research purposes and to guide policy, practice and action were identified. Analysis revealed eight categories of challenges. These challenges covered a range of domains. These included infrastructure-related issues in the Accessibility and Remote Access categories, which addressed issues such as the number of steps to be taken in applying for access to data, and the time it takes, as well as challenges linked to the requirement to access data on site. Other categories addressed more user-focused issues, including the Understanding and Skill, Expectations and Standards, Resistance and Trust, and Awareness categories, in which issues were raised in relation to lack of knowledge of the NILS database, resistance to innovative and creative uses of data, lack of statistical skills and a mismatch in expectations versus what is delivered. The remaining categories, Funding, Research and Policy, and Policy Challenges, raise issues around the lack of funding for projects, a lack of co-designed research between researchers and policy-makers and a lack of buy-in from public bodies and/or government departments. Figure 6 presents a sample of challenges within each category. The full set of challenges can be found in Additional file 3: Appendix C, Table S1.

### Analysis of NILS resources

Upon reviewing NILS resources – the data dictionary, the past projects section of the website and the application form – a range of challenges were noted with presentation, understanding and decision-making using the website, and understanding the data using the data dictionary and completing the application process using the data request form.

#### Data dictionary challenges

Participants generated 39 data dictionary challenges (Additional file 4: Appendix D, Table 1) across four themes. The Finding Variables theme addresses challenges in data dictionary navigation, including the inability to search for specific variables, and the lack of key theme tags.

The Identifying and Understanding Variables theme addresses challenges associated with lack of clarity regarding distinctions between variables, a lack of variable context provided and the need for linkage back to the original data collection form to ascertain how responses were coded.

The Usability theme included challenges associated with using Microsoft Access, which is not familiar to most users, the inability to automatically connect the data dictionary to the application form (for example, when selecting variables for inclusion) and the need for more usable, modern formats.

Finally, Integration and Linkages addressed the lack of integration across NILS, NIMS, Administrative Data Research Centre (ADRC) and Business Services Organization (BSO), as well as the need for greater coherence with the census, and the lack of provision of lists of possible linked data sources.

#### Website challenges

Participants generated 13 challenges in relation to the past projects section of the website (Additional file 4: Appendix D, Table 2). The majority of these relate to access to information about the projects, such as a lack of detailed information on the variables used, a lack of a key word search option for project review, a lack of consistency in the detail of project summaries and the fact that projects are not grouped together by category. The above were described as barriers for potential NILS users, who may be searching for past projects which are relevant to a project they are planning, and information on the variables used in past projects.

#### Application form challenges

In relation to the application form, participants generated a total of nine challenges (Additional file 4: Appendix D, Table 3). These challenges covered a range of issues, including the need for the application form to be updated and made more accessible and user-friendly, the need for prompts and guidance that an online application form could provide, the need for standardization across related longitudinal studies (LSs) to remove the burden on users from different regions and the need for additional clarity, definition and support in completing the form, including specific guidance on the health focus requirement that is central to all NILS projects.

### Analysis of user needs

Using SBD methods, workshop participants also identified specific user needs across three domain areas:

- Information needs
- Collaborative and decision-making needs
- Training and analysis needs.

**Fig. 7:**
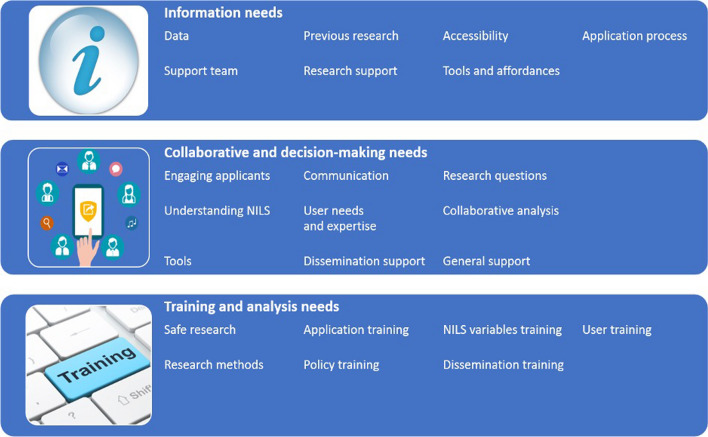
Infographic depicting a sample of key challenges within information, collaborative and decision-making and training and analysis needs

Figure 7 presents an overview of needs categories generated across domains. In summary, seven categories of information needs, nine categories of collaborative and decision-making needs and seven categories of training and analysis needs were identified. Further below is an analysis of needs for each domain, separately, and reasons for these needs.

### Information needs

**Fig. 8:**
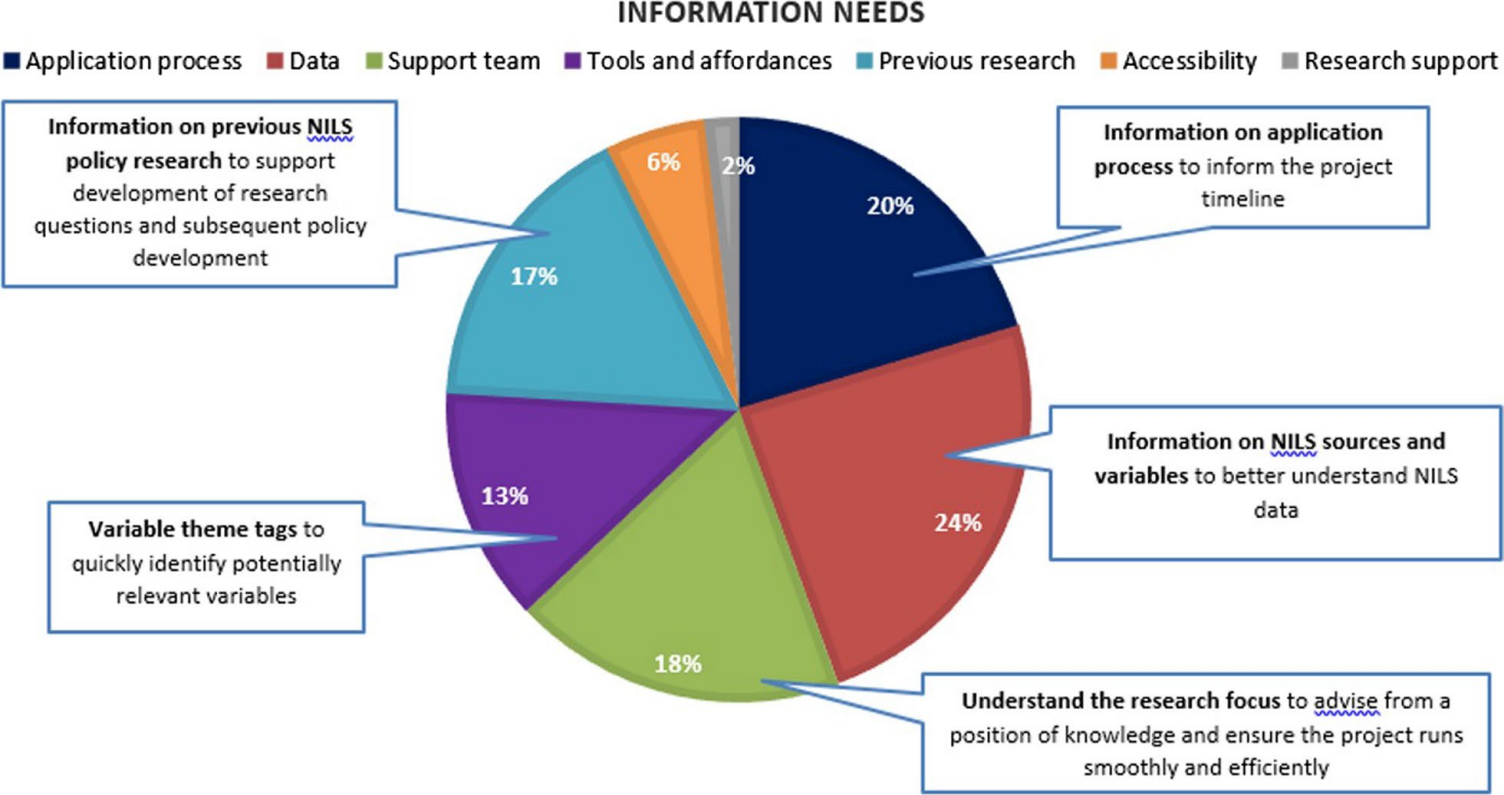
Pie chart categorizing information needs

Approximately one quarter of all information needs fell into the Data Needs category (24%). The next largest category was Application Process needs (20%), followed by Support Team (18%), Previous Research (17%), Tools and Affordances (13%), Accessibility (6%) and Research Support needs (2%; Fig. 8). For example, in relation to Data, participants highlighted the need for information about the sources of NILS, as well as more information about the variables themselves. This was suggested as important in gaining a better understanding of NILS data. Participants also raised the need for more information in relation to the application process, as this is necessary to factor into project timelines. Participants noted that access to information about previous policy-related NILS projects would support the development of future research questions, as well as policy development. To review the full set of needs within each category, please see Additional file 5: Appendix E, Table 1.

### Collaborative and decision-making needs

**Fig. 9:**
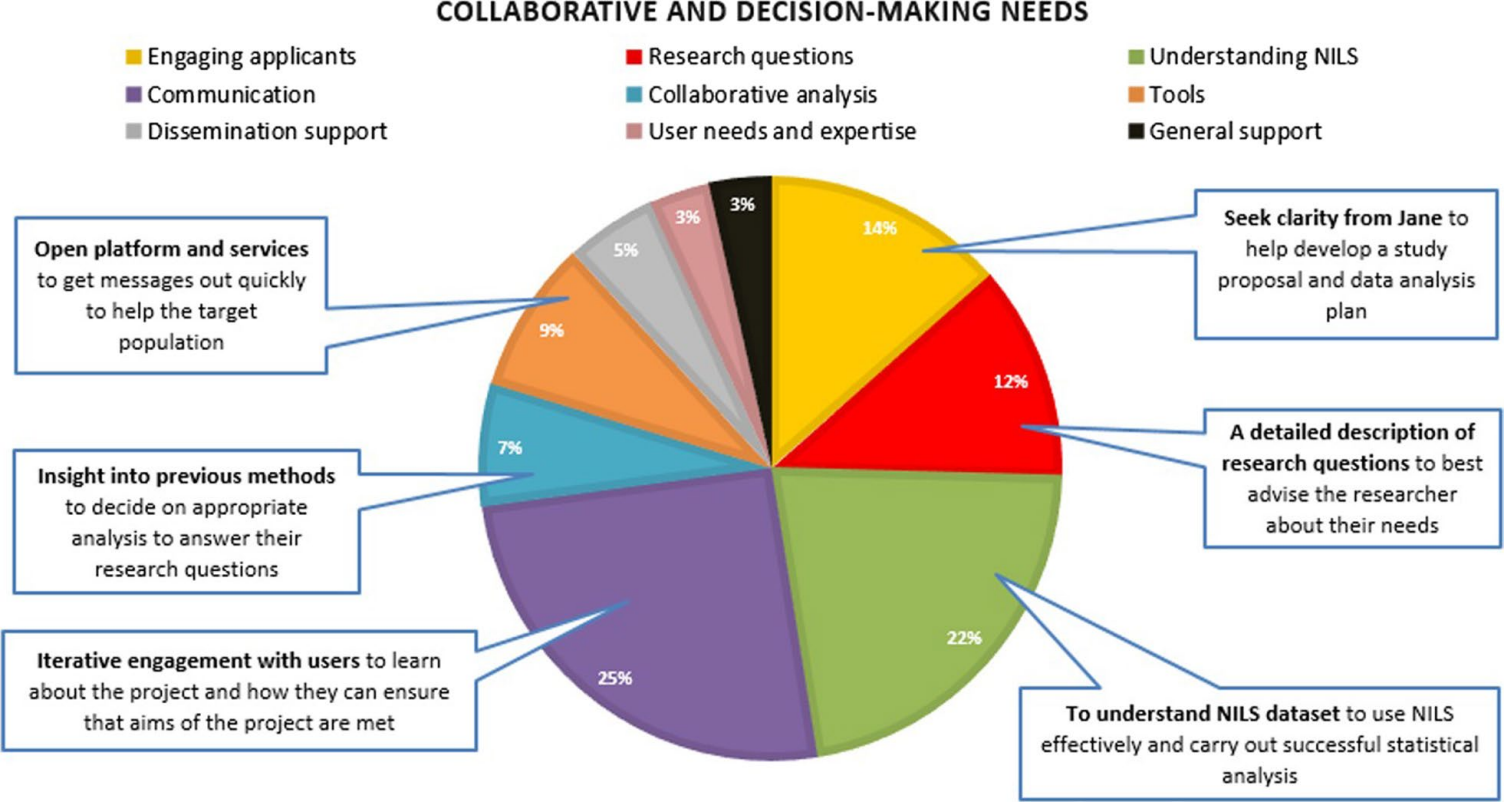
Pie chart categorizing collaborative and decision-making needs

The largest percentage of collaborative and decision-making needs fell into the Communication category (25%). This was followed by Understanding NILS (22%), Engaging Applicants (14%), Research Question (12%), Tools (9%), Collaborative Analysis (7%), Dissemination Support (5%), User Needs and Expertise (3%) and General Support needs (3%; Fig. 9). Among the Communication needs was the call for iterative engagement with users to enable the RSU to learn about the aims of the proposed project and help guide the researchers in this process. Participants also highlighted, in the Understanding NILS category, the need for the collaborative development of a shared understanding between researchers and the RSU, such that NILS data can be used effectively. Figure 8 below provides some further examples of identified needs, with the full set of categorized collaborative and decision-making needs presented in Additional file 5: Appendix E, Table 2.

### Training and analysis needs

**Fig. 10:**
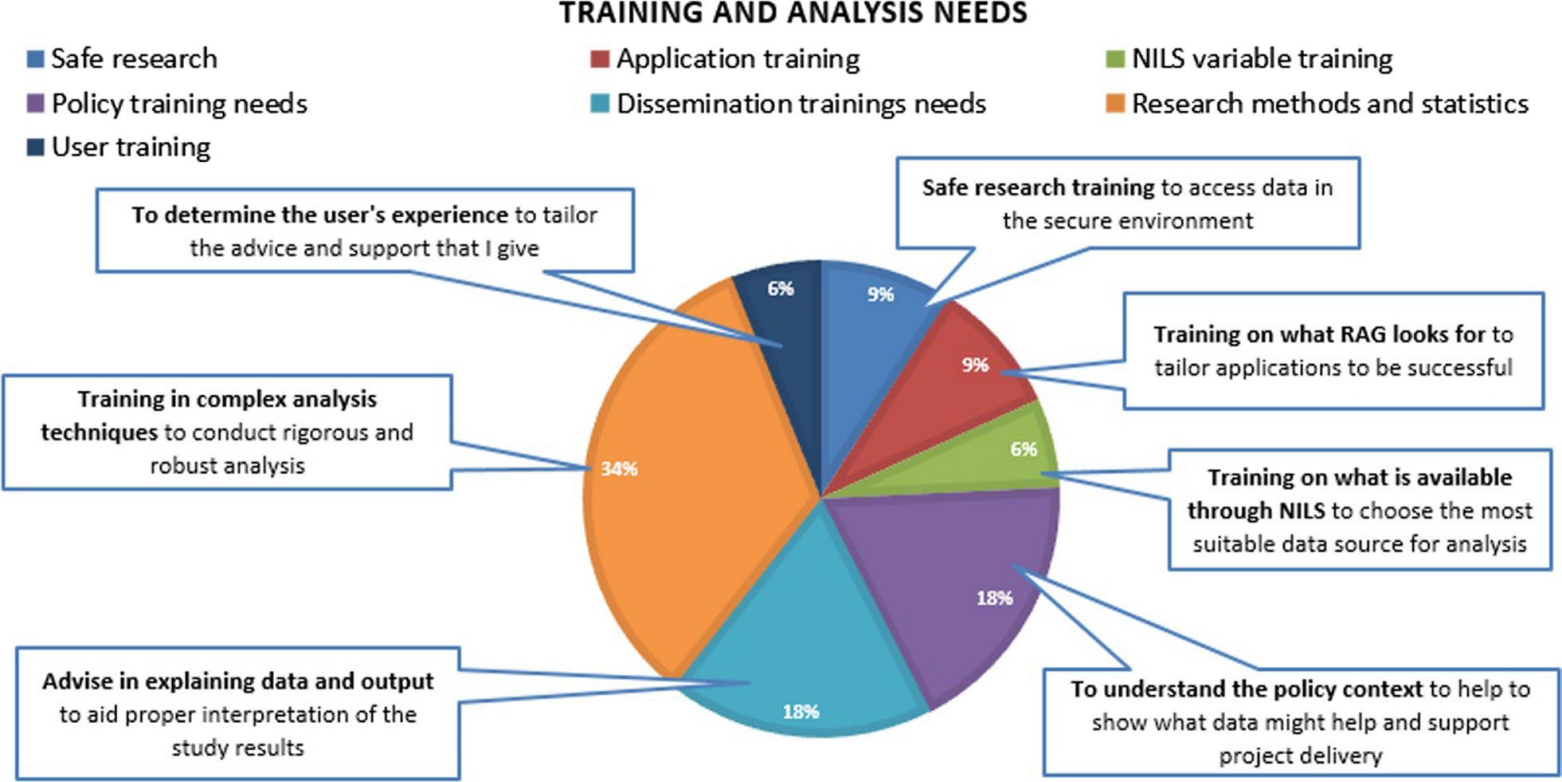
Pie chart categorizing training and analysis needs

Over one third of all training and analysis needs fell into the Research Methods and Statistics category (34%; Fig. 10). In this category, participants highlighted the need for training in complex analysis techniques, to help them to conduct rigorous and robust analysis, as well as training in the interpretation of complex analysis. The Training and Analysis need also included categories around Dissemination Training and Policy Training. These categories included reference to needs such as advice and support in interpreting and explaining the data and output from studies, as well as the need for training in contextualizing data for the purposes of policy-related studies. Notably, participants also identified training needs on the part of the RSU members, as well as researchers. Participants also highlighted a need to provide training to NILS RSU staff on how to advise and support users in writing a successful application. It was proposed that this may involve a review of previous successful applications, as well as those which were unsuccessful for minor reasons, or reasons which could have been fixed or avoided before the application was submitted. It was proposed that this may allow NILS RSU staff to give specific advice to help users avoid common pitfalls identified in previous unsuccessful applications. To review the full set of categorized training and analysis needs, see Additional file 5: Appendix E, Table 3.

### Implementation

The RSU infrastructure design team reviewed the outputs of the CI session and engaged in a dialogue in relation to the potential impact and feasibility of design changes aligned with user needs and the types of challenges users are currently experiencing in accessing, understanding and using NILS data. The set of actions implemented by the RSU team are outlined in Table 1.

**Table 1:** Actions implemented by the RSU team in response to the areas for development highlighted within the CI report

Development area	Action
Accessibility: understanding of data infrastructure	A redesigned website was launched under an independent URL (www.nils.ac.uk). The primary target for the website is new researchers interested in utilizing NILS. Video overviews and step-by-step guides to researcher accreditation and project planning are to the forefront and presented in non-technical language. The new website also includes field representations of the topics and thematic spread of NILS projects, using material developed for the CI workshop. The medium-term aspiration is to have a dedicated policy-maker space and dashboard through which all relevant NILS findings can be accessed in key point format.
Accessibility: understanding role of NILS within NI data landscape	Signposting role and conference: NILS RSU advises prospective users of alternative options to NILS, depending on the proposed project, timescale and requirements (for example, data analysis focus, granularity). The academic team organized the Belfast Big Data Day in collaboration with Belfast City Council at Belfast City Hall, and with other data providers (including Ordinance Survey and Labour Force Survey). Whilst the pandemic hampered follow-up from this event, successful online seminars have bridged the gap in the interim, particularly “The potential use of NILS for Covid research”, which saw an invited panel of academics and those working in government policy talk about the future evidence gaps.
Accessibility: understanding data/metadata	Metadata: The new website details classes and sources of data, including individual and household-level census demographics, health records and environmental risks.
Accessibility of data	Remote access: Both major Northern Ireland (NI) universities who provide the majority of users have entered into new agreements with government data custodians across the UK and now have Assured Organizational Connectivity agreements. These recognize the universities as trusted and secure institutions and permit access from campus-based university computers. Long-term, if extended to NI census data, this could remove a logistical barrier to preparation of NILS data for analysis.
	Use of synthetic data: As of 2022, NISRA have made synthetic data files available, which contain variables with the same names, codings and univariate distributions as would be found in a full NILS population. A carefully prepared non-disclosive dataset, stripped of private information but with capacity for building analytical models, has been launched as a learning resource on the website together with complimentary tutorials and information.
User needs: information; collaborative decision-making	User voice structures and research community: Previously active groups were merged under the banner of the Northern Ireland Administrative Data Researcher’s User Forum. The forum facilitates discussion space involving researchers and data technicians, with five meetings every year. Researchers are represented on the NILS Steering Committee by the forum chair.
Public need: clear communication of NILS Findings	Briefings project: Funding was secured for a two-strand project. First, a one-day specialist training day was provided to researchers, with the instructor assisting researchers in the creation of policy briefs based on existing work. Subsequently, a part-time research assistant was recruited for weekly sessions in which to further develop these briefs and devise a search strategy for maintaining an up-to-date record of live policy consultations and parliamentary calls for expert input in the UK and beyond. Case study files of how each policy brief could be amended to fit particular calls were also created.

## Discussion

Governments around the world are increasingly focused on developing collaborative data infrastructures that support engagement of citizens, researchers and public administrators in the development of shared understanding of societal issues and solutions to a range of public policy challenges [1, 4]. The design of government data infrastructures is important in efforts to make data accessible, understandable and usable [7, 8, 10], but a variety of technical, innovation and social barriers have been identified which highlight the need for ongoing and iterative redesign of existing data infrastructures [11–17, 19]. Understanding the needs of stakeholders and data users is important in this context, as it helps to develop specific requirements that can feed into the planning and development work undertaken by data infrastructure design teams.

The NILS RSU team has identified the need to improve their data infrastructure not only to support understanding and primary use of complex national data but also to increase the dissemination and secondary impact of research based on these data. This infrastructure design work requires careful attention to, and coordination of, multiple components, including the existing and evolving data available for use, policies and procedures in accessing and using the data, researcher and administrative support, awareness building and communication, creating networks and partnerships, as well as capacity-building. The NILS RSU team recognize that this infrastructure design challenge necessitates a stakeholder-engaged and systematic approach to design work. The CI process described in this paper, in identifying key challenges to accessing, understanding and using NILS data and the specific information, collaboration and decision-making needs of users, provided a collaborative and constructive foundation for ongoing design work.

Notably, many of the challenges faced in the context of the NILS RSU infrastructure design and delivery are broadly consistent with those identified in the wider literature. For example, issues of accessibility and remote access are well-recognized [13, 17], and in the case of the NILS, CI participants identified a range of challenges, including: the number of steps in the data application process, requirements associated with applying for access to data, the time it takes, and the need to access data on-site. Similarly, consistent with literature documenting challenges associated with Understanding and Skill, Expectations and Standards, Resistance and Trust, and Awareness [12, 13, 17], CI participants in the NILS RSU design project noted challenges, such as lack of knowledge of the database, resistance to innovative and creative uses of data, lack of statistical skills and a mismatch in expectations versus what is delivered. Finally, the types of challenges faced by the NILS under the categories of Funding, Research and Policy, and Policy Challenges issues, including the lack of funding for projects, a lack of co-designed research between researchers and policy-makers, and a lack of buy-in from public bodies and/or government departments, are reminiscent of similar challenges faced in other jurisdictions, such as issues of cost and funding, as well as reluctance or lack of prioritization by local or national government organizations [14, 16].

To identify specific areas to be addressed as part of a larger project of infrastructure design, participants engaged in the CI process identified a range of user needs that covered the full spectrum of NILS-related activity, including the application process, accessing and understanding data and analysing NILS data. The needs and requirements of prospective users warrant careful consideration in the context of NILS design, given the broader trend of open data uptake and usage lagging behind open data availability [10, 34]. Consistent with other examinations of open data infrastructure and usage [7, 8, 10], the need for quality data and metadata was raised as an important requirement for NILS users. Analysis of CI results also highlighted a range of needs in relation to functionality, training and support, which have been highlighted to be key priorities for open data infrastructures across multiple local and national projects. Notably, following a survey analysis of Open Government Data Infrastructures (OGDIs) across 52 countries, Zuiderwijk and De Reuver concluded that even more influential than issues of data quality and metadata is the type and range of functionality available to users, as well as the types of supports provided to users. Following this analysis, Zuiderwijk and De Reuver suggested that a key focus of data infrastructure design should include tools to enable data analysis, interpretation and visualization, as well as training support, and contact points that coordinate and deliver user support services (for example, help desks) [35].

The CI process used by the NILS RSU team proved to be a key input at a critical point in the evolution of NILS. It informed the framing of a new programme for the continued funding and development of NILS infrastructure, highlighting key constituencies of stakeholders and potential users who could be better served through enhancements in communication and engagement. The COVID pandemic saw secure room closure and increased demands on public service resources, causing several NILS projects to be paused. Conversely, the resources consolidated within the new NILS website provided a new window of opportunity to learn about the NILS data and its potential uses. Furthermore, the COVID experience has bolstered the case for several NILS data access enhancements proposed in the course of the CI workshop, including facilities for data access via secure remote connections. Additionally, use of synthetic data for preparing data and analysis protocols was invaluable and has been permanently made available online.

Participatory design of data infrastructure has continued through collaborative conversation with Belfast City Council along with a range of data custodians that can work together to address Northern Ireland’s most pressing issues: sustainable planning of built environments, educational inequalities, employment and mental ill-health. Furthermore, the convening of a Northern Ireland Administrative Data Researcher’s User Forum represents an attempt to bring researchers into an ongoing consultative dialogue with data custodians. It also serves to bring together researchers using a range of facilities, including NILS, Health and Social Care (HSC) records curated by the HSC Honest Broker Service, and bespoke linkage projects drawing from wider administrative records.

The Collective Intelligence workshop marked an important staging post in thinking across the boundaries of different disciplines and types of records. With increasing trust among data custodians in the procedures, systems and research culture around the NILS, a greater amount of data at small-area level concerning, for example, migration, education and the physical environment have been made available to researchers.

### Key challenges and limitations

When it comes to addressing societal challenges and supporting deliberative and participatory approaches to policy development and project implementation, ongoing stakeholder-engaged CI design of government data infrastructures is important to enhance and sustain informed engagement and shared understanding. However, it has been noted that national administrative data are seldom, if ever, “research-ready” in their raw form, and the labour of matching and combining separate data sources into standardized presentations of variables is often challenging [36]. McGrath-Lone et al. identified key attributes of a research-ready data resource, in particular, that it is accessible, broad, curated and documented. Even where these conditions are broadly in place, researchers can encounter trade-offs, for example, between data accessibility versus breadth and precision [37]. In the case of national census data, the number of topics which can be addressed through a nationwide survey is necessarily limited, as is the depth to which those topics are investigated. Some data will be protected from secondary use out of concern for citizens’ privacy. This may be controlled at the access stage, through monitoring of output, reporting or a combination. Therefore, a group or social intersection which is so small as to risk identifying individuals which fit those combined descriptions is likely to be removed from the dataset or aggregated into a larger group to protect privacy. Similarly, the degree of geographical specificity which can be reported is subject to risk assessment and control.

These limitations give rise to a wider challenge for the dissemination and use of national linked data and related open data platforms, namely that the expectations of potential users may be misaligned with the knowledge that can be gained from these resources. Furthermore, findings from datasets which rely upon proxy measures and where there is limited scope for follow-up analysis may ultimately be of limited interest to policy-makers and practitioners. A way to circumvent these difficulties is through involving stakeholders in identifying analytical priorities and new data infrastructure affordances that enhance the use of data. For this to be successful, engagement should be underpinned by mutual respect for stakeholder expertise and for the responsibilities of analysts and system designers.

## Conclusion

Our primary objective to improve government data infrastructure and to increase dissemination and the impact of research based on data was a complex and multifaceted challenge due to the number of stakeholders involved and their often conflicting perspectives. In the absence of a systematic approach to reconciling these viewpoints, building a shared vision for system design is difficult.

As the NILS RSU team look to the upcoming Census 2021 linkage, the CI report has been pivotal in highlighting how RSU can work collaboratively with users to maximize the potential of this data, in terms of forming multidisciplinary networks to ensure the research is addressing gaps in policy and in the literature and providing academic support and resources to attract new researchers. The current CI design project has highlighted the importance of engagement and collaboration with users to quickly adapt to changing needs, which has been highlighted by the pandemic. The changes already implemented by the RSU have improved the infrastructure immeasurably and have gone some way towards our vision of NILS as an invaluable data resource within the Northern Ireland data landscape.

### Supplementary Information

**Additional file 1: Appendix A.** See for more detail on collective intelligence methods.

**Additional file 2: Appendix B.** Scenarios – Two of the scenarios from the ideawriting session.

**Additional file 3: Appendix C.** Full set of challenges to accessing, understanding and using NILS data.

**Additional file 4: Appendix D.** Full set of NILS Resources Challenges.

**Additional file 5: Appendix E.** Full set of needs.

## Data Availability

All data generated or analysed during this study are included in this published article [and its Additional file].

## Abbreviations

*ADRC*: Administrative Data Research Centre
*BSO*: Business Services Organization
*CI*: Collective intelligence
*DLP*: Distinct linkage project
*ESRC*: Economic and Social Research Council
*HSC*: Health and social care
*IM*: Interactive management
*NILS*: Northern Ireland Longitudinal Study
*NIMS*: Northern Ireland Mortality Study
*NISRA*: Northern Ireland Statistics and Research Agency
*RSU*: Research Support Unit
*SBD*: Scenario-based design
