# Supplementary material for: Using collective intelligence methods to improve government data infrastructures and promote the use of complex data: The example of the Northern Ireland Longitudinal Study
Source: Health Res Policy Syst. 2023 Dec 18;21:134. doi: 10.1186/s12961-023-01070-x (PMC10726592; doi:10.1186/s12961-023-01070-x)
Supplement: Supplementary file 1 — Additional file 1: Appendix A. See for more detail on collective intelligence methods. [file 12961_2023_1070_MOESM1_ESM.pdf]

Overview of Collective Intelligence approach

The *Collective Intelligence* approach carefully delineates content and process roles, assigning to participants responsibility for contributing ideas and to the facilitator responsibility for choosing and implementing selected methodologies for generating, clarifying, structuring, interpreting, and amending ideas. Emphasis is given to balancing behavioral and technical demands of group work (Broome & Chen, 1992) while honoring design laws concerning variety, parsimony, and saliency (Ashby, 1958; Boulding, 1966; Miller, 1956). CI has been applied in a variety of situations to accomplish many different goals, including mediating peacebuilding in protracted conflicts (Broome, 2006), developing a national well-being measurement framework (Hogan et al., 2015), understanding and overcoming barriers to the design of personalised nutrition products and services for older adults (Hogan et al., 2017), and Designing an e-learning tool to support health practitioners caring for patients taking multiple medications (Hanlon et al, 2021).

In a typical CI session, a group of participants who are knowledgeable about a particular situation engage in (a) developing an understanding of the situation they face, (b) establishing a collective basis for thinking about their future, and (c) producing a framework for effective action. In the process of moving through these phases, group members can develop a greater sense of teamwork and gain new communication and information-processing skills.

CI utilises a carefully selected set of methodologies, matched to the phase of group interaction and the requirements of the situation. The most common methodologies are the nominal group technique, ideawriting, interpretive structural modeling, field representations, and, scenario-based design and user stories. The first two methodologies are primarily employed for the purpose of generating ideas that are then structured using one or more of the

latter methodologies. The current study used NGT, ideawriting, scenario-based design (SBD) and agile stories, but not Interpretive structural modeling (ISM).

The *nominal group technique* (NGT; Delbeq, Van De Ven, & Gustafson, 1975) is a method that allows individual ideas to be pooled, and is best used in situations in which uncertainty and disagreements exist about the nature of possible ideas. NGT involves five steps: (a) presentation of a stimulus question to participants; (b) silent generation of ideas in writing by each participant working alone; (c) “round-robin” presentation of ideas by participants, with recording on flipchart by the facilitator of these ideas and posting of the flipchart paper on walls surrounding the group; (d) serial discussion of the listed ideas by participants for sole purpose of clarifying their meaning (i.e., no evaluation of ideas is allowed at this point); and (e) implementation of a closed voting process in which each participant is asked to select and rank five ideas from the list, with the results compiled and displayed for review by the group.

Ideawriting (Warfield, 1994) is a method that utilizes relatively small groups of 4-6 persons each, formed by dividing a larger group into several working teams, for the purpose of developing ideas and exploring the meaning of those ideas through open discussion. Ideawriting involves five steps: (a) presentation of a stimulus question to participants; (b) silent generation of ideas in writing by each participant working alone; (c) exchange of written sheets of ideas among all group members, with opportunity for individuals to add ideas as they read others’ papers; (e) discussion and clarification of unique ideas; and (f) an oral report of the ideas generated by each working group in a plenary session. In this plenary session, duplicate ideas across the working groups are eliminated from the set and new ideas (if any) are added; the resulting set of ideas is then ready for use in the next stage of the group’s work, which might involve one or more of the following methodologies.

The methodology used to gather *user-level requirements* combines *CI* with *scenario-based design (SBD)* (Rosson & Carroll, 2002) and *agile user story development* (Cohn, 2004). The SBD framework (Rosson & Carroll, 2002) describes an iterative approach to interactive systems design and analysis, and encourages a reasoning process about people using technology and about finding trade- offs throughout development, including trade-offs between the potential impact of design decisions and the feasibility of the design options. Scenario-based design uses stories, or 'scenarios', at an early point in the development process to describe how a user might interact with a system. "A user interaction is a sketch of use. It is intended to vividly capture the essence of an interaction design, much as a two-dimensional, paper-and-pencil sketch captures the essence of a physical design" (Rosson & Carroll, 2002, p. 1032, italics in original). Instead of focussing on defining system operations, SBD, similar to other user-centred approaches, focusses on "how people will use a system to accomplish work tasks and other activities" (Rosson & Carroll, 2002, p. 1032).

According to Rosson and Carroll (2002) scenarios are stories that consist of:

- A setting or situation state;
- One or more actors with personal motivations, knowledge, and capabilities;
- Various tools and objects that the actors encounter and manipulate; and
- A sequence of actions and events that lead to an outcome that includes the goals, plans and reactions of the people taking part.

User stories can be derived from more complex scenarios and are also often used in the development of software. They are simple stories that describe a discrete and specific functionality that a user wants from the software (Cohn, 2004). Scenarios and user stories are popular in interactive system design because they facilitate communication amongst members of the design team in relation to usage possibilities and the problems and issues that arise for different stakeholders. Simple scenarios are relatively easy to write and it takes only a little

more effort to enrich the scenario with a rough sketch or storyboard. When designers are working through ideas, they often wish to make progress quickly, so that they can obtain feedback from stakeholders and the design team and continue to refine their ideas. Scenario and user story development provides a valuable source of data to work with in this context. In our CI scenario-based design and user story approach, short user stories generated by participants are generated in the form:

As User Type _______, I want ______, so that I can ____

Interpretive structural modeling (ISM; Warfield, 1994) is a computer-assisted methodology that helps a group to identify relationships among ideas and to impose structure on those ideas to help manage the complexity of the issue. Specifically, the ISM software utilizes mathematical algorithms that minimize the number of queries necessary for exploring relationships among a set of ideas (see Warfield, 1976). ISM can be used to develop several types of structures depicting the relationships among a set of ideas, including influence structures (e.g., "supports" or "aggravates"), priority structures (e.g., "is more important than" or "should be learned before") and categorizations of ideas (e.g., "belongs in the same category with"). The five steps of ISM are: (a) identification and clarification of a list of ideas (using a method such as NGT or ideawriting); (b) identification and clarification of a "relational question" for exploring relationships among ideas (e.g., "Does idea A support idea B?," "Is idea A of higher priority than B?," or "Does idea A belong in the same category with idea B?"); (c) development of a structural map by using the relational question to explore connections between pairs of ideas (see below); (d) display and discussion of the map by the group; and (e) amendment to the map by the group, if needed.

In the third step of developing a structural map, questions are generated by the ISM software and are projected onto a screen located in front of the group. The questions take the following form:

"Does idea A relate in X manner to idea B?"

"A" and "B" are pairs of ideas from the list developed by participants in the first step of ISM and the question of whether they "relate in X manner" is the statement identified in the second step.

For example, if a group is developing an influence structure with problem statements, the question might read:

"Does problem A significantly aggravate problem B?"

Using the ISM methodology, the group engages in discussion about this relational question and a vote is taken to determine the group's judgment about the relationship. A "yes" vote is entered in the ISM software by the computer operator if a majority of the participants see a significant relationship between the pair of ideas; otherwise, a "no" vote is entered. Another pair of ideas is then projected on the screen in front of participants, another discussion is held, and a vote is taken. This process is continued until the relationships between all necessary pairs of ideas have been explored. The ISM software then provides to the facilitator the information from which a structural map can be constructed, showing the result of the group's series of judgments about pairs of ideas. The length of time required to complete discussion of all necessary pairs of ideas depends on the total number of ideas in the set, but, generally, the process requires between 3-5 hours of group deliberation. The number of necessary queries also depends on the total number of ideas in the set, but the ISM software is able to infer during the structuring process an average of approximately 70-80% of the judgments involved in relating the complete set of ideas.

The influence structuring work conducted with ISM can be considered an activity in "mapping perceptions" of the group members. Participants are given the opportunity to explore connections and links between ideas in ways that probably would have gone undetected without such structuring work. ISM can, thus, provide participants with useful insights into the relationships between ideas and it generates a product, a structural map of those relationships, which can guide their thinking as they design potential solutions.

Field representation (Warfield & Cardenas, 1995) organizes ideas in a way that allows a large amount of information to be worked with effectively. There are different types of field representations that are useful for different types of applications, but, typically, a field representation portrays a significant amount of information organized in a form that (a) is appropriate for use in making decisions and (b) maintains an ongoing, visible record of intermediate decision making en route to a final portrayal of the total set of choices that has been made. A field representation shows a set of categories and the members of each of those categories. When appropriate, the group might engage in a structuring process (using ISM) to sequence the categories according to agreed-on criteria.

The portrayal of choices in the field representation technique constitutes a *profile representation*. In constructing a profile, a group examines the first category of the field and chooses elements from that category. Each choice is represented graphically by drawing a line from the bullet in front of a selected element down to a "tie line," a continuous line drawn at the base of the graphic, beneath the full category set. After all choices are made, the selected elements are connected to the tie line; all elements that have not been selected remain unconnected. In this way, the viewer is presented with a graphical portrayal of both selected items and the full set of items considered for inclusion in the final product.

References

Ackoff, R. L. (1981). *Creating the corporate future: Plan or be planned for*. New York: John Wiley and Sons.

Alberts, H. (1992, March). *Acquisition: Past, present and future*. Paper presented at the meeting of the Institute of Management Sciences and Operations Research Society, Orlando, FL.

Argyris, C. (1982). *Reasoning, learning, and action: Individual and organizational*. San Francisco: Jossey-Bass.

Ashby, W. R. (1958). Requisite variety and its implications for the control of complex systems. *Cybernetica*, *1*(2), 1-17.

Attalides, M. A. (1979). *Cyprus: Nationalism and international politics*. New York: St. Martin's Press.

Bahcheli, T. (1990). *Greek-Turkish relations since 1955*. Boulder, CO: Westview Press.

Boulding, K. E. (1966). *The impact of the social sciences*. New Brunswick, NJ: Rutgers University Press.

Broome, B. J. (1993). Managing differences in conflict situations. In D. J. D. Sandole & H. van der Merwe (Eds.), *Conflict resolution theory and practice: Integration and application* (pp. 97-111). Manchester, United Kingdom: Manchester University Press.

Broome, B. J. (1995a). Collective design of the future: Structural analysis of tribal vision statements. *American Indian Quarterly*, *19*, 205-228.

Broome, B. (1995b). The role of facilitated group process in community-based planning and design: Promoting greater participation in Comanche tribal governance. In L. R. Frey (Ed.), *Innovations in group facilitation: Applications in natural settings* (pp. 27-52). Cresskill, NJ: Hampton Press.

Broome, B. J. (1997). Designing a collective approach to peace: Interactive design and problem-solving workshops with Greek-Cypriot and Turkish-Cypriot communities in Cyprus. *International Negotiation, 2,* 381-407.

Broome, B. J. (1998). Overview of conflict resolution activities in Cyprus: Their contribution to the peace process. *Cyprus Review*, *10*, 47-66.

Broome, B. J. (1999, September). Greek and Turkish Cypriot university students have more in common than expected. *Washington Report on Middle East Affairs*, pp. 82-84.

Broome, B. J. (2001). Participatory planning and design in a protracted conflict situation: Applications with citizen peace-building groups in Cyprus. *Systems Research and Behavioral Science*, *18*, 1-9.

Broome, B. J., & Chen, M. (1992). Guidelines for computer-assisted group problem-solving: Meeting the challenges of complex issues. *Small Group Research*, *23,* 216-236.

Broome, B. J., & Christakis, A. N. (1988). A culturally-sensitive approach to tribal governance issue management. *International Journal of Intercultural Relations*, *12,* 107-123.

Broome, B. J., & Cromer, I. L. (1991). Strategic planning for tribal economic development: A culturally appropriate model for consensus building. *International Journal of Conflict Management*, *2*, 217-234.

Broome, B. J., & Fulbright, L. (1995). A multi-stage influence model of barriers to group problem solving. *Small Group Research*, *26,* 25-55.

Broome, B. J., & Keever, D. B. (1989). Next generation group facilitation: Proposed principles. *Management Communication Quarterly, 3*, 107-127.

Broome, B. J., & Murray, J. S. (2002). Improving third-party decisions at choice points: A Cyprus case study. *Negotiation Journal*, *18*, 75-98.

Bolukbashi, S. (1998). The Cyprus dispute and the United Nations: Peaceful non-settlement between 1954 and 1996. *International Journal of Middle Eastern Studies, 30*, 411-434.

Burton, J. W. (1969). *Conflict and communication: The use of controlled communication in international relation*s. London: MacMillan.

Calotychos, V. (Ed.). (1998). *Cyprus and its people: Nation, identity, and experience in an unimaginable community, 1955-1997*. Boulder, CO: Westview Press.

Christakis, A. N. (1985). High-technology participative design: The space-based laser. *Proceedings of the Society for General Systems Research*, *2*, 925-933.

Christakis, A. N. (1987). Systems profile: The Club of Rome revisited. *Systems Research*, *4*, 53-58.

Cleveland, H. (1973). The decision makers. *Center Magazine*, *6*(5), 9-18.

Coke, J. G., & Moore, C. M. (1981). Coping with a budgetary crisis: Helping a city council decide where expenditure cuts should be made. In S. W. Burks & J. F. Wolf (Eds.), *Building city council leadership skills: A casebook of models and methods* (pp. 72-85). Washington, DC: National League of Cities.

Cronen, V. E. (1995). Practical theory and the tasks ahead for social approaches to communication. In W. Leeds-Hurwitz (Ed.), *Social approaches to interpersonal communication* (pp. 217-242). New York: Guilford Press.

Craig, R. T. (1989). Communication as a practical discipline. In B. Dervin, L. Grossberg, B. J. O'Keefe, & E. Wartella (Eds.), *Rethinking communication: Volume I: Paradigm issues* (pp. 97-122). Newbury Park, CA: Sage.

Craig, R. T., & Tracy, K. (1995). Grounded practical theory: The case of intellectual discussion. *Communication Theory*, *5*, 248-272.

Deal, T. E., & Kennedy, A. A. (1982). *Corporate cultures: The rites and rituals of corporate life*. Reading, MA: Addison-Wesley.

Delbeq, A. L., Van De Ven, A. H., & Gustafson, D. H. (1975). *Group techniques for program planning: A guide to nominal group and Delphi processes*. Glenview, IL: Scott, Foresman.

Diamond, L., & Fisher, R. J. (1995). Integrating conflict resolution training and consultation: A Cyprus example. *Negotiation Journal*, *11*, 287-301.

Doob, L. W. (1974). A Cyprus workshop: An exercise in intervention methodology. *Journal of Social Psychology*, *94*, 161-178.

Feeg, R. (1988). Forum of the future of pediatric nursing: Looking toward the 21st century. *Pediatric Nursing*, *14,* 393-396.

Fisher, R. J. (1992). *Peace building for Cyprus: Report on a conflict analysis workshop, June 1991*. Ottawa: Canadian Institute for International Peace and Security.

Fisher, R. J. (1997). *Interactive conflict resolution*. Syracuse, NY: Syracuse University Press.

Fisher, R. J., & Keashly, L. (1988). Third-party interventions in intergroup conflict: Consultation is NOT mediation. *Negotiation Journal*, *4,* 381-393.

Folger, J., Poole, M. S., & Stutman, R. K. (2000). *Working through conflict: Strategies for relationships, groups and organizations* (4th ed.). New York: Addison Wesley Longman.

Frey, L. R. (Ed.). (1995). *Innovations in group facilitation: Applications in natural settings*. Cresskill, NJ: Hampton Press.

Friedman, P. G. (1989). Upstream facilitation: A proactive approach to managing problem-solving groups. *Management Communication Quarterly*, *3,* 33-50.

Hadjipavlou-Trigeorgis, M. (1993). Unofficial inter-communal contacts and their contribution to peace-building in conflict societies: The case of Cyprus. *Cyprus Review*, *5*(2), 68-87.

Hadjipavlou-Trigeorgis, M. (1998). Different relationships to the land: Personal narratives, political implications and future possibilities in Cyprus. In V. Calotychos (Ed.), *Cyprus and its people: Nation, identity, and experience in an unimaginable community, 1955-1997* (pp. 251-276). Boulder, CO: Westview Press.

Hirokawa, R. Y., & Gouran, D. S. (1989). Facilitation of group communication: A critique of prior research and an agenda for future research. *Management Communication Quarterly, 3*, 71-92.

Hitchens, C. (1997). *Hostage to history: Cyprus from the Ottomans to Kissinger*. New York: Noonday Press.

Joseph, J. S. (1997). *Cyprus: Ethnic conflict and international politics: From independence to the threshold of the European Union*. New York: St. Martin's Press.

Keever, D. B. (1989, April). *Cultural complexities in the participative design of a computer-based organization information system*. Paper presented at the International Conference on Support, Society and Culture: Mutual Uses of Cybernetics and Science, Amsterdam, The Netherlands.

Kemeny, J. (1980). Saving American democracy: The lesson of Three Mile Island. *Technology Review, 83*(7), 64-75.

Koumoulides, J. T. A. (Ed.). (1986). *Cyprus in transition: 1960-1985*. London: Trigraph.

Markides, K. C. (1977). *The rise and fall of the Cyprus Republic*. New Haven, CT: Yale University Press.

McDonald, P. (1990). *Group support technologies*. Cambridge, MA: U.S. Department of Transportation, Federal Aviation Administration.

Miller, G. A. (1956). The magical number seven, plus or minus two: Some limits on our capacity for processing information. *Psychology Review*, *63*, 81-97.

Mirbagheri, F. (1998). *Cyprus and international peacemaking*. New York: Routledge.

Mitchell, C. R. (1981). *Peacemaking and the consultant's role*. Farnborough, Hampshire, England: Gower.

O'Malley, B., & Craig, I. (1999). *The Cyprus conspiracy: America, espionage and the Turkish invasion*. New York: I. B. Tauris.

Parrish-Sprowl, J. (2003). Indexing the Polish transformation: The case of ECO-S from a bona fide group perspective. In L. R. Frey (Ed.), *Group communication in context: Studies of bona fide groups* (2nd ed., pp. 291-305). Mahwah, NJ: Lawrence Erlbaum.

Poole, M. S. (1983). Decision development in small groups II: A study of multiple sequences in decision making. *Communication Monographs, 50*, 206-232.

Poole, M. S. (1985). Tasks and interaction sequences: A theory of coherence in group decision-making interaction. In R. L. Street & J. N. Cappella (Eds.), *Sequence and pattern in communicative behavior* (pp. 206-224). London: Edward Arnold.

Poole, M. S., & Doelger, J. A. (1986). Developmental processes in group decision-making. In R. Y. Hirokawa & M. S. Poole (Eds.), *Communication and group decision making* (pp. 35-62). Newbury Park, CA: Sage.

Poole, M. S., & Roth, J. (1989). Decision development in small groups IV: A typology of group decision paths. *Human Communication Research, 15*, 323-356.

Richmond, O. P. (1998). *Mediating in Cyprus: The Cypriot communities and the United Nations*. London: Frank Cass.

Rittel, H., & Webber, M. (1974). Dilemmas in a general theory of planning. *DMG-DRS Journal, 8*, 31-39.

Sato, T. (1979). Determination of hierarchical networks of instructional units using the ISM method. *Educational Technology Research, 3*, 67-75.

Savvides, P. K. (2002). *Cyprus at the gate of the European Union: Scenarios, challenges, and prospects.* Athens, Greece: Hellenic Foundation for European and Foreign Policy.

Simon, H. A. (1960). *The new science of management decisions*. New York: Harper & Row.

Simon, H. A. (1974). How big is a chunk? *Science*, *183*, 482-488.

Stearns, M. (1992). *Entangled allies: U.S. policy toward Greece, Turkey, and Cyprus*. New York: Council on Foreign Relations Press.

Stoddard, P. H. (1986). An experiment in track two diplomacy. In D. B. Bendahmane & J. W. McDonald, Jr. (Eds.), *Perspectives on negotiations: Four case studies and interpretations* (pp. 139-143). Washington, DC: Center for the Study of Foreign Affairs, Foreign Service Institute, U.S. Department of State.

Talbot, P. (1977). The Cyprus seminar. In M. R. Berman & J. E. Johnson (Eds.), *Unofficial diplomats* (pp. 159-167). New York: Columbia University Press.

Theophanous, A. (1996). *The political economy of a federal Cyprus*. Nicosia, Cyprus: Intercollege Press.

Volkan, V. D. (1979). *Cyprus—war and adaptation: A psychoanalytic history of two ethnic groups in conflict*. Charlottesville: University Press of Virginia.

Warfield, J. N. (1976). *Societal systems: Planning, policy, and complexity*. New York: Wiley.

Warfield, J. N. (1994). *A science of generic design: Managing complexity through systems design* (2nd ed.). Salinas, CA: Intersystems.

Warfield, J. N., & Cardenas, A. R. (1994). *A handbook of interactive management* (2nd ed.). Ames: Iowa State University Press.
