# Supplementary material for: Using collective intelligence methods to improve government data infrastructures and promote the use of complex data: The example of the Northern Ireland Longitudinal Study
Source: Health Res Policy Syst. 2023 Dec 18;21:134. doi: 10.1186/s12961-023-01070-x (PMC10726592; doi:10.1186/s12961-023-01070-x)
Supplement: Supplementary file 2 — Additional file 2: Appendix B. Scenarios – Two of the scenarios from the ideawriting session. [file 12961_2023_1070_MOESM2_ESM.pdf]

Appendix B - Scenarios

First scenario

Jane is a senior **public administrator** in a Belfast. Jane is helping to prepare a new plan to promote local community and economic development in Belfast and wants to explore how the NILS data might be used to facilitate a wider engagement between her research team and local community leaders to facilitate bottom up community building. Jane is particularly interested in social mobility (i.e., changes in socioecomonic status over time) and changes in patterns of social inequality across neighbourhoods and school districts in Belfast, and key factors that may influence social mobility and inequality. Jane believes that an understanding of these dynamics will help her research team to engage in collaborative discussions with communities and inform policies and projects across regions. Jane, who has little or no understanding of statistical analysis, works directly with **Joe**, who is **a senior data analyst** who has just moved from London to Belfast. Joe is unfamiliar with the NILS data, so he logs onto the NILS website to learn more about previous studies that have been conducted on social mobility and inequality. Having reviewed the pattern of results across **previous NILS projects** using the **interactive project analysis tools**, Joe decides to move closer to the data and examines the new interactive **NILS data insight tool**, which provides a rich way of understanding the NILS data and ways in which it can be **linked to other datasets** to inform specific research and policy issues. Joe also examines the new **interactive data request form** that supports efficient data request submissions. Joe struggles to understand some key details and so he contacts **Fiona and Frank**, who lead the **NILS research support team** and provide guidance, training, collaborative support to research teams, public administrators, and communities who wish to develop knowledge grounded in the analysis and application of NILS data. In a deeply engaging and rewarding iterative learning process, Jane works with Joe and the NILS team over a period 8 weeks to meet a key report deadline, and moves from there to negotiate and plan activities with other public administrators and community leaders in her local authority public administration offices. She wants both citizens and her colleagues in the local community development group to have some flexibility in the way they draw upon data and information when working together to develop community projects, and she appreciates the iterative engagement with the NILS team. Jane is very passionate about promoting local community and economic development in Belfast and across Northern Ireland and she wants a platform and set of services that will help her do good work.

Second scenario

Sarah is a senior **community leader** in a Belfast. **Sarah** is helping to prepare a new plan to promote employment in Belfast and wants to explore how the NILS data might be used to facilitate a wider engagement between local community leaders and public administrators to facilitate bottom up community building. **Sarah** is particularly interested in mental health in this context. More specifically, she is interested in the links between unemployment, social mobility (i.e., changes in socioecomonic status over time) and mental health across neighbourhoods and school districts in Belfast, and key factors that may influence unemployment and inequality. **Sarah** works with **Sam**, a public administrator, as well as two master's students, **Paul and Joanne** to explore this issue. Sam believes that an understanding of these dynamics will help them to engage in collaborative discussions with communities and inform policies and projects across regions. **Sam**, who has little or no understanding of statistical analysis, works directly with **Paul and Joanne**, who have some experience in data analysis on their master's course. However, **Paul and Joanne** are unfamiliar with the NILS data, so they log onto the NILS website to learn more about previous studies that have been conducted on social mobility, unemployment and mental health. Having reviewed the pattern of results across **previous NILS projects** using the **interactive project analysis tools**, the team decide to move closer to the data and examine the new interactive **NILS data insight tool**, which provides a rich way of understanding the NILS data and ways in which it can be **linked to other datasets** to inform specific research and policy issues. **Paul** also examines the new **interactive data request form** that supports efficient data request submissions. The team struggle to understand some key details and so they contact **Fiona and Frank**, who lead the **NILS research support team** and provide guidance, training, collaborative support to research teams, public administrators, and communities who wish to develop knowledge grounded in the analysis and application of NILS data. In a deeply engaging and rewarding iterative learning process, **Sarah, Sam, Paul and Joanne** work with the NILS team over a period 8 weeks to meet a key report deadline, and move from there to negotiate and plan activities with other public administrators and community leaders in the local authority public administration offices. **Sarah** wants both citizens and her colleagues in the local community development group to have some flexibility in the way they draw upon data and information when working together to develop community projects, and she appreciates the iterative engagement with the NILS team. **Sarah** is very passionate about promoting mental health awareness and employment opportunities in Belfast and across Northern Ireland and she wants a platform and set of services that will help her do good work.
