# Supplementary material for: Using collective intelligence methods to improve government data infrastructures and promote the use of complex data: The example of the Northern Ireland Longitudinal Study
Source: Health Res Policy Syst. 2023 Dec 18;21:134. doi: 10.1186/s12961-023-01070-x (PMC10726592; doi:10.1186/s12961-023-01070-x)
Supplement: Supplementary file 3 — Additional file 3: Appendix C. Full set of challenges to accessing, understanding and using NILS data. [file 12961_2023_1070_MOESM3_ESM.pdf]

Appendix C - Full set of challenges to accessing, understanding, and using NILS data

**Awareness**
• Lack of knowledge that NILS exists and how to access/utilise it
• Confusion over different sources e.g. ADRC v NILS
• Limited access to information on Distinct Linkage Projects – e.g. detailed information on what sort of information has been linked to the NILS in existing projects, and what other data can be linked in future projects. There is information on the BSO HBS website, but it not clear how to access this from the RSU website, and it is not clear whether all the data listed on BSO website is linkable to NILS and how tight the constraints on such linkages would be
**Resistance and trust**
• Lack of trust in researchers to use the data appropriately
• Resistance to creative and innovative uses of data e.g. data linkage, synthetic data, remote access
**Policy challenges**
• Lack of transparency around how policy is created, who creates it, and how researchers can directly influence its creation
• Lack of buy in from some public bodies/government departments
• Northern Ireland has limited policymaking levers, restricting the ability of research findings to be translated into policy proposals
• Inadequate understanding of policy context and players
**Expectations and standards**
• Delivery falls short of expectations generated by those promoting the use of NILS
**Understanding and skills**
• Lack of understanding of structure and content of datasets
• Lack of understanding of limitations of datasets
• Fear of large datasets
• Lack of appropriate statistical skills
**Accessibility**
• Too many hoops to jump through to get access to data

• Process takes too long
• Inability to access data quickly
• Infrequency of RAG meetings for project approval (currently every two months) may be a barrier to some researchers – particularly those on short-term contracts – undertaking a NILS project
**Remote access**
• Unwillingness to work in a remote location
• Inability to use NILS outside Northern Ireland would be a large barrier for projects set up by researchers based in other parts of the UK (I am aware there is an eDatashield facility, but this seems to be restricted to multi-LS projects)
• Inability to access data quickly
• Inability to access data wherever (i.e., have to travel to use data)
**Research and policy**
• Researchers and policymakers often have different views of what constitutes interesting/worthwhile research. Researchers (in academia) will typically only invest their time in projects that offer results capable of being published in academic journals
• Researchers are not incentivised to undertake policy-based work (e.g. producing a policy guidance document based on research findings) unless there is a possibility for journal publishing or financial grant awards
• Inadequate consideration of potential to impact practice in initial project development
• A problem wider than the NILS itself is that a long time lag exists between setting up and completing a project, which probably limits the viability of regular policy-researcher interface meetings
• Lack of co-designed research questions with policymakers/groups etc.
• There is a superstition among researchers about disseminating material which hasn't been peer reviewed or published – people think this risks publication or criticism which creates a lag in sharing knowledge and gaining impact. Northern Ireland Life and Times (NILT) are a model for regular dissemination, data updates and newsletters. NILS research briefs aren't sufficiently regular or bought into among users.

Funding
• Very few NILS projects have their own funding sources. People aren’t paid or funded to be NILS community members. For most, it represents a smaller percentage of their time, available energy and output
• Time delays with accessing data are often absorbed into the project costings
• The NILS exists within a competitive funding environment, but ESRC are a central funder to many resources. To my mind, projects have more of a competitive mindset than one of collaboration and signposting. Part of our jobs as NILS community members should be to recommend datasets which aren’t within NILS where these are more appropriate to the objectives at hand (e.g. UK Data Archive).
