# Supplementary material for: Using collective intelligence methods to improve government data infrastructures and promote the use of complex data: The example of the Northern Ireland Longitudinal Study
Source: Health Res Policy Syst. 2023 Dec 18;21:134. doi: 10.1186/s12961-023-01070-x (PMC10726592; doi:10.1186/s12961-023-01070-x)
Supplement: Supplementary file 4 — Additional file 4: Appendix D. Full set of NILS Resources Challenges. [file 12961_2023_1070_MOESM4_ESM.pdf]

Appendix D – Full set of NILS Resources Challenges

Table 1. Data dictionary challenges

**Finding variables**
• Lack of search function
• Inability to search specific variables
• Lack of search function within individual tables on access database
• Lack of key theme tags
• It is quite an arduous task to find variables
• Overwhelming amount of information/tables. It is difficult to know where to start to look for variables and if they are suitable for the research project
• Difficult to navigate data dictionary (not easy to find what you're looking for)
**Identifying and understanding variables**
• Need for researcher to have a very clear idea of the project before starting to look at the data dictionary
• Need for reference between recorded variables
• Need for linkage back to the original form – what was ticked and how was this coded?
• Lack of ability to search for responses
• Need for linkage out to relevant census table
• Lack of available examples e.g. items on programme for government, departmental work packages
• Unclear how certain variables differ
• There is a lack of clarity of the level of duplication where some variables are concerned
• Need to understand the difference between data sources even if they appear to be providing the same data/variable
• Difficult to identify the variable without knowledge of all options within variables
• Lack of metadata
• Problems with identifying which variables to use. Some are detailed with a more aggregated version available but difficult to see differences between variables using the data dictionary.

• Lack of context when using the data dictionary only to select variables. Requirement for background information on datasets which can be found in metadata document.
• It would be useful to get information on coverage of each variable i.e. years of available data
• Hard to visualise some of the data and so can't get a sense of what it looks like e.g. pollution data
• Lack of information about all available variables in a simple way
• It is a bit ambiguous in places with use of "other"
• Inability to identify cases of "other" e.g. no occupation group for artists. Are there no artist in Northern Ireland? Are there insufficient numbers for a category?
• It is unclear whether free-text responses can be identified – if they collect the data, can you request it?
• Absence of detail re. health conditions e.g. yes/no response to illness – but no detail of which illness
**Usability**
• Data dictionary could be in a more up to date format which might make navigating variables more easy
• Need to improve software – MS Access is not common to users
• The data dictionary is in a quite inaccessible format. It is difficult to manipulate/get the information required easily
• It is hard to read the full variable information – the boxes could be bigger
• There is a need for more difficult manual or help prompts when completing the variable list
• Lack of experience in using the data dictionary – would need time and support in familiarising with it?
• Inability to automatically connect the data dictionary to the application form
**Integration and linkages**
• The project may be amenable to a DLP project but it is not immediately apparent if the outstanding data would be available for linkage (education-specific variables)
• Lack of integration (e.g. NILS, NIMS, ADRC, BSO)
• Need for greater coherence with census
• Need for list of datasets which have been or could be linked to NILS to help answer research question

• List of possible linked data sources of information required is unavailable

Table 2. Past projects challenges

Problems with protecting IP but trying to provide enough information on the website to allow researchers to establish whether their project is novel
May be useful to show a list of variables being used or those that have been used in current/past projects – sometimes unclear exactly what was studied
The most closely aligned project is still currently underway and so is only a powerpoint presentation available for guidance as to what has been done to date using the NILS. There is not enough data in the presentation to answer all our questions
Some enquiries would require the input from service support staff as there was not enough information available on the NILS website
Lack of key word search for projects
Projects not grouped together by category
Unattractive website
Difficult to view research findings in order to build upon these
Lack of detailed information on the variables used or outcomes of the projects listed
Lack of structure around the display of projects e.g. could be structured in a diagram by topic area
Lack of links to outputs for previous projects and sometimes no access to papers
Inability to search at the item level (e.g. past project on volunteering – but cannot find higher order category for data selection)
Lack of consistency in project summaries

Table 3. Application form challenges

Need for an online form
Absence of standardisation across LSs
Lack of a central web location for cross LS projects
Inadequate information in the legal framework
Need for support in identifying the population

Need for guidance on project titles (catchy? How long?)
Need for guidance on possible duration (re: funding period)
Need for note regarding safe researcher status
Need for high-level of clarity with regard to the effects of including certain sub-groups of variables – need to know clearly which variables and sub-groups of variables are relevant
