# Supplementary material for: Using collective intelligence methods to improve government data infrastructures and promote the use of complex data: The example of the Northern Ireland Longitudinal Study
Source: Health Res Policy Syst. 2023 Dec 18;21:134. doi: 10.1186/s12961-023-01070-x (PMC10726592; doi:10.1186/s12961-023-01070-x)
Supplement: Supplementary file 5 — Additional file 5: Appendix E. Full set of needs. [file 12961_2023_1070_MOESM5_ESM.pdf]

Appendix E

Table 1. Information needs -- information design supporting discovery and understanding, relevant data and past findings, tools supporting navigation of relevant information

As (User X),	I want ……	, so that I can …..
**Paul & Joanne**	Information on variables relating to mental health, unemployment, socioeconomic status and prescribing data	Decide on variables to include in analyses
**Fiona & Frank**	To understand the focus of the research and timescales	Advise from a position of knowledge and ensure the project runs smoothly and efficiently.
**Sam**	To know what other data (e.g. other UK regions or datasets) can address the questions in this project	Present findings to stakeholders with the context of how NI is similar/different, link to policy initiatives elsewhere and verify findings independently
**Paul & Joanne**	Information on the data included in the NILS, any gaps or quality issues	Select the most appropriate data to analyse and don't waste time looking at poor data that won't produce trustworthy results
**Fiona and frank**	Clear information on what kind of output is needed from the project and what it should achieve	Help design a project plan and ensure all variables are included in the application that may be needed
**Sarah**	Know what previous research has been carried out in this area	She is not duplicating already completed research and can focus analysis on adding to this
**Sam**	To know what other researchers have investigated in this area	Look up their recommendations in prior outputs invite their input.
**Paul and Joanne**	To know who in the NILS community has looked at mobility and mental health	Ascertain the best measures to use, avoid any pitfalls and be aware of any limitation
**Paul & Joanne**	Information on previous research around mental health mobility and socioeconomic status	Narrow focus of research in appropriate direction

**Sarah**	To know about previous research in the area	Identify knowledge gaps and see what is still needed to influence change
**Paul & Joanne**	Information on what relevant data is available in NILS and what previous projects have found in this research area	Plan the analysis to answer the research question based on available NILS data
**Sarah**	Updates as analysis unfolds on the big picture of employment and mental health and early findings	Ask specific follow up questions such as any area level variations in effect.
**Sarah**	Information on findings of previous research	Direct research in appropriate direction
**Fiona**	To have quick reference guide to the variables the team are requesting	Anticipate possible issues at output level such as low cell counts and advise the researchers
**Sarah**	Information on the application process & any guidance on how to complete this	Submit an application form quickly and in a format that can be reviewed by RAG and approved.
**Paul and Joanne**	Interactive sample size calculations	Plan analysis and know what power I have to answer questions
**Fiona and Frank**	Detailed information on what the project is about and what data is required – interactive form	Ensure that they give appropriate advice to project team
**All**	A progress report stage online – e.g. you are on the stage completed	Evaluate the stage in progress of application for data
**Paul/Joanne**	Variable theme tags	Quickly identify potentially relevant variables
**Paul & Joanne**	NILS data insight tools	Understand the specificity of NILS data
**Sam and Sarah**	Research findings based on NILS analysis	Inform discussions with communities and inform service provision
**Sam**	To have a review of actions/potential actions (electronic)	So that I can easily see the impact/potential impact of research in policy
**Fiona & Frank**	Clear understandings of the needs of everyone	Use of NILS dataset for maximum use and effectiveness
**Paul**	Interactive data request form	Put in place an efficient data request submission
**Fiona and frank**	A wide range of help facilities available online	Use NILS members teams more efficiently e.g. stretching data
**Sam**	Statistical data analysis	Create meaningful use of data to engage collaborative discussions with communities

**Sarah**	Database of plain English summaries of past findings and uniform template	Quickly determine the current evidence base
**Paul & Joanne**	Information on structure and content of NILS	Make informed decision about data required for project and to complete data form
**Sarah**	Interactive information sharing platform	Disseminate findings of research to a wide audience
**Sarah**	A suite of tools	Showcase data in different ways for policy needs
**Sam**	Information on what data and types of analysis are appropriate for research questions	Receive appropriate results to negotiate and plan activities with other stakeholders.
**Jane**	A search function which allows me to see previous research in my area of interest (e.g., interactive word cloud)	Understand topic area better and help refine research question
**Joe**	Information on NILS sources and variables	Better understand NILS data
**Fiona and Frank**	Information on required timescales for receipt of data at point of application	Deliver the requested dataset in a timely manner
**Jane**	The findings to be displayed clearly and concisely	Understand exactly what the results of the analysis show so that she can develop policy
**Joe**	To know what variables are held in the NILS and what is the unique identifier in common with NILS and other relevant datasets	Understand how the linking of datasets is achieved and which sets may be of use
**Jane**	Information on the needs and expectations of local community leaders	Deliver a plan to improve social mobility and reduce inequality
**Jane**	To know what information is held in the NILS	determine whether it can be used to increase my understanding of the relevant issues on social mobility in my communities of interest
**Joe**	Clear instruction via the website on how to apply for access to NILS data	Develop and submit a research application within tight timescales
**Joe**	An easy way of understanding the underlying distributions of data	Determine whether the sample size is enough to support a robust analysis

**Jane and Joe**	An online tool that I can use to provide an overview of the available data	Identify which data I need to answer my question
**Fiona and Frank**	Information on the needs and expectations of community leaders	Deliver workshops/presentation on potential of NILS
**Jane**	To have the data analysis supplied to me in an understandable format	Understand what the statistical analysis means to me this will allow me to convey the information to my audiences such as the community leaders and policy makers
**Joe**	To review previous studies using NILS data and speak to Fiona and frank – NILS data insight tool	Understand the data and how it can be useful to help achieve overall objective
**Fiona and frank**	To ascertain the information needs of jane and joe	Provide the appropriate level of guidance, training, and collaborative support to both jane and joe
**Jane**	Information on social mobility and inequality in local communities	Set targets and objectives for improvement
**Joe**	Information on whether a similar project has already been carried out and findings made public	Draw on existing research where possible
**Joe**	Online list of alternative, linkable data sources together with estimated acquisition time	Understand whether further linkage is possible within project timeline
**Joe**	Easily accessible information on previous NILS projects and outcomes	To inform research questions
**Fiona and frank**	Live access to joe and jane's table of selected variables	Support analysis decisions
**Jane**	Information on previous NILS research and how it impacted policy	To support development of research questions and subsequent policy development
**Joe**	Access to similar projects and the tables of variables used	Design a novel study
**Joe**	Information on the application process	To inform project timeline
**Fiona and frank**	Clear information of the project aims	Help to produce the application

**Joe**	If data are accessible only in secure environment, which statistical software exist?	To help plan for data exploration and analysis
**Joe**	Review past findings	To avoid ‘reinventing the wheel’ their data analysis should provide fresh insights
**Fiona and frank**	Knowledge of factors associated with efficient approval rates	To make approval process as efficient as possible
**Jane**	Easy navigation of all past projects on community development	Design my study
**Joe**	Clear info on how the process works	Have realistic expectations
**Jane**	Info on average length of time for all processes required	Plan my research
**Joe**	Access to dummy/synthetic data	To understand the data included and how it can be used
**Jane**	Access to previous research	Design my study effectively
**Jane**	Past findings	To see if questions have already been answered
**Joe**	NILS data insight tool	Understand the data that are available and linkages
**Fiona/frank**	Study the project proposal prepared by jane and joe	To decide the extent of support they require
**Joe**	To be able to view similar past projects	Build upon the research and get a clear picture
**Fiona and frank**	Interactive data request form	To advise on which variables/alternatives to request

Table 2. Collaboration and Decision-making needs -- what types of collaboration and decision-making tools, methods, and communication processes are needed?

As (User X),	I want ……	, so that I can …..
**Sarah**	Someone to translate signals from the data into clear ideas and action points	Leverage engagement with local data to instigate change in my community
**Paul & Joanne**	Help in understanding the context of NILS variables	Decide on appropriate analysis
**Sam**	A clear tie in from the analysis to NILS to current policies	Make the case for continued investment into employment assistance and into research to inform policies
**Fiona & frank**	A research question from Sarah and her team	Provide the best guidance and advice on the NILS data variables in order to plan a clear project to answer their question
**Sarah**	To demonstrate that what I see on the ground in communities is affecting the population at large	Make a case for investment which will save money in the long run (e.g. cutting down mental health treatment) and improve people's lives)
**Sarah**	Identify the most appropriate groups to work with in the community	Ensure meaningful research which will have a major impact in the community
**Fiona**	To work together with Sarah and her team to help create a clear project making use of the NILS data	Contribute to research in this area and make an impact on employment opportunities and mental health awareness
**Sarah**	Guidance on how to publish research finding	Reach appropriate audience groups
**Sam**	Translation of findings in layman's terms	Decide how findings impact on direction of future policy.
**Sarah**	Information on whether NILS has the data needed	Explore links between mental health, unemployment and social mobility
**Paul & Joanne**	Work closely with experts in statistical analysis	Carry out robust analysis which produces trusted results
**Fiona & Frank**	Support from colleagues and leadership, i.e. time in my day	Work closely with the research team to help deliver their report in a tight timeline

**Joanne**	Help understanding the NILS data and previous research in this area	Analyse the links between social mobility and unemployment
**Fiona & Frank**	Understanding of Sarah and Sam's exact needs in terms of policy answers	Provide guidance in terms of data available and links with research
**Sarah**	Collaborate with others to identify the best method of disseminating the results	Have as large an impact as possible and promote future research in this area and use of the NILS
**Paul**	Clear guidance on the value and meaning of different NILS variables	Figure out the best variables to use to capture work and employment
**Sam**	To see if patterns of employment and mental health differ in Belfast versus wider NI	Recommend similar approaches based on the project with Sarah and spread lessons beyond Belfast
**Paul & Joanne**	Insight into methods of analysis used in previous research	Decide on appropriate analysis to answer their research questions
**Paul**	To have detailed guidance on completing the form	Get access to the data I need more quickly and correctly
**Sam**	Analysis of datasets	Use findings to understand dynamics between unemployment, social mobility etc. to inform collaborative discussions
**Paul & Joanne**	To understand NILS data as a unique data set	Use NILS effectively and carry out successful statistical analysis
**Sam**	Clear understanding of the project needs from Sarah	Communicate those to Paul and Joanne
**Paul & Joanne**	Information on data variables	To inform the type of analysis
**Sarah**	A platform and set of services	Assess mental health, unemployment and social mobility
**Fiona and Frank**	Iterative engagement process with Sarah Sam Paul and Joanne	Learn about the project and how they can ensure that aims of the project are met
**Sarah**	Open platform and services	To get message out quickly to help the target population
**Sam**	Others to support the statistical analysis and data aspect of project	Inform policy and create outcomes
**Sarah**	Evidence	Develop community building
**Fiona**	Research questions and project plans, application forms etc.	Assist users with project completion

**Frank**	To understand the level of expertise and needs of the group	Support them in their experience using NILS
**Sam**	To be able to effectively communicate my questions to Paul and Joanne	Can get more details and informative answers from statistics
**Paul/Joanne**	Data	Complete master's degree
**Sam**	Collaborative discussions with communities	To inform policies and projects across regions
**Fiona & Frank**	To understand level of expertise of team	Can offer the best level of support to ensure safe and accurate use of data
**Paul**	Interactive data request form	Effectively and efficiently request the data that he needs to the analysis
**Sarah**	To have public and professional engagement	Make sure all perspectives are considered and delivered on
**Sam**	A statistical understand of the data	Understand the dynamics and share these with Sarah and wider policy context
**Sarah**	Being able to understand the research findings	So that I can identify concrete policy actions that may lead to effective change
**Sarah**	Reassurance that NILS will provide appropriate evidence	Judge project feasibility
**Paul and Joanne**	NILS data insight tool	Make informed decision about the data they need and to help complete the data request form efficiently
**Jane**	To facilitate wider encouragement with local community leaders	Prepare a new plan to promote local community and economic development
**Joe**	Help from NILS support team	Understand the NILS data
**Fiona and Frank**	To engage with Jane, Joe and local community leaders	Provide guidance and support
**Joe**	To engage with Fiona and Frank	Understand the NILS data and other key details in order to determine if the research questions can be answered
**Joe**	To communicate with jane and the community leaders	Understand the potential outcomes of his analysis and findings
**Fiona and frank**	Engage with jane	To understand nature of flexible relationship with NILS team

**Joe**	A detailed picture of the variables that would enable me to answer my questions	Narrow down my research questions
**Jane**	An accessible description of the data and the research output	So that she can relay knowledge to community contacts
**Fiona & frank**	A detailed description of the research questions and the output that jane and joe want	Best advise the researcher about their needs
**Jane**	To understand the key factors that may influence social mobility and inequality	Engage with communities and inform policies and projects across neighborhoods and school districts
**Joe**	To help provide the appropriate statistical support to interrogate the NILS data [if appropriate]	Determine the statistical relationships/patterns that jane is interesting in to allow her to engage with the community groups in an informed and meaningful manner
**Fiona and Frank**	To help local community leaders understand what NILS can do for them	Help jane and joe to deliver a plan for them to improve social mobility and reduce inequality
**Joe**	To understand the NILS dataset and other data sources	Advise on/develop an analysis plan to meet Jane's needs
**Jane**	To collaborate with Joe	Utilize his analytical skills in order to deliver a robust report
**Fiona and frank**	To help joe improve his understanding of NILS	He can help jane to prepare her new plan for local communities
**Jane**	To collaborate with Fiona and Frank	Develop a framework for flexible engagement with the NILS team in order to meet the needs of community groups/local government
**Jane**	To form discussions with Fiona and frank	With the view of gaining flexibility in how the data may be used in the future
**Fiona and Frank**	To understand exactly what jane and joe are hoping to achieve in their use of the data	Provide the correct and most useful guidance and collaborative support to the project
**Jane**	To prepare a new plan to promote local community and economic development	Improve social mobility and reduce inequality
**Joe**	To better understand NILS	Analyse the data to help jane deliver her new plan
**Jane**	To understand the influencing factors on social mobility and changes in social inequality in	Have informed discussions with those community representatives

	the community areas she serves	and inform policies and projects in her jurisdiction
**Joe**	To improve understanding of NILS data	Help jane
**Fiona and Frank**	To engage with Jane and Joe	Understand the broader research questions and advise on suitability and availability of data to meet their needs
**Jane**	To work with joe to provide information in an understandable format	Design services which improve the life circumstances of the community she serves and engage with policy makers to make real change
**Joe**	To understand the NILS process and usage	Solve the research issue and get results which are useful
**Fiona/frank**	To understand jane and joes question	Support best practice analysis
**Jane**	To develop a platform and set of services to enable promotion of local and community and economic development	Succeed in my engagement plan to work towards increasing bottom up community building
**Jane**	Further discussions with Fiona and frank	To know if her research questions could be answered with existing data
**Fiona and frank**	Pay more attention to janes work	To understand the application of their data in solving local problems
**Jane**	Seek opinion of community members	To find out if they really care about the research
**Joe**	Clear information on the linkages with NILS and possibility of other linked datasets	To identify the best data to inform the research question
**Jane**	Information about statistical analysis	Be able to process data
**Jane**	Communicate decision/plan to relevant stakeholders	So that they can understand the relevance of the project
**NILS team [RAG]**	Understand social /health impact of research project	Approve the project
**Joe**	Understand the use of NILS and linking datasets	Have a data rich analysis that is useful and informative
**Fiona and frank**	An efficient approval process	Complete the approval in 8 weeks and meet the report deadline
**Joe**	To use NILS effectively to carry out research	Gain meaningful insights

**Jane**	To understand policy vision and priorities of NILS	Get approval to access the data
**Jane**	To collaborate with a data analyst [joe]	To have the necessary skills to analyse the NILS data and answer my research question
**Fiona/frank**	To assist the researchers to get approval for their project	Enable policy to be developed for communities
**Jane**	To see past results using similar variables	Generate hypotheses and design study/interventions in community
**Jane**	To promote local community and development	Better service provision for Belfast
**Jane**	Direct communication with joe	Design study to examine impact of geography on mobility
**Joe**	Seek clarity from jane	To help develop a study proposal and data analysis plan
**Joe**	More knowledge about the data available in NILS	Decide how best to answer my research questions
**Joe**	More knowledge about data available in other datasets	Explore potential data linkage project
**Joe**	To understand the process of applying for access to NILS	Apply to NILS successfully
**Joe**	Further discussion with jane to understand research objectives	So that he can choose relevant statistical test/method of analysis
**Jane**	Flexibility in the way in which people draw upon data	Ensure collaborative approach to develop community projects
**Jane**	To inform future policy and projects across neighborhoods and school districts	Promote bottom up community building that is informed by evidence
**Fiona and frank**	To support researchers in making their applications	Enable them to carry out meaningful research and inform policy makers

Table 3. Training and analysis needs -- what types of advice, supports, training programme content, procedures and methods are needed?

As (User X),	I want ……	, so that I can …..
**Paul & Joanne**	More training on analysis methodologies/software	Analyse the data and avoid wasting time
**All**	Safe researcher training	Access data in the secure environment
**All**	Safe researcher training	See intermediate outputs and contribute to write-up
**Paul & Joanne**	Further training on statistical analysis techniques	Quickly run robust analysis to produce trustworthy results
**Sarah**	Advice on how to complete the application form	Complete the form quickly and have the proposal accepted as soon as possible
**Fiona & Frank**	Clear guidance on rules around spatial or geographically sensitive analysis	Treat outputs appropriately and guide users on a safe level of output and presentation
**Sam**	Training in interpretation of complex analysis such as spatial modelling and interaction effects	Monitor outputs from this project and adjust recommendations and action plan accordingly
**Paul**	My hand held (somewhat) when running syntax in Stata and R	Be sure I'll get analysis done on time and that it says what I think it says
**Sarah**	Visual explanation of the analysis	Clearly present the key takeaways to various audiences
**Sarah/Paul/Joanne**	Clear guidance on the rules for outputs and dissemination	Avoid breaching anonymity or associated sanctions
**Sarah**	Advice from local communities on the specific policy questions they would like answered	Carry out meaningful research which will have an impact for the communities I am working with
**Paul & Joanne**	Ongoing statistical and software training	Analyse the data correctly and efficiently and interpret findings correctly
**Fiona & Frank**	In depth training on the datasets and variables available, the source and quality of these	Advice to the best of their ability on which

		data/variables can answer the research question
**Sarah**	Advice on what kinds of results she needs to make her research impactful	So her research project can make an impact in the community
**Frank & Fiona**	Researchers to be safe users of the NILS data	Ensure the data owners and data is not being misused
**Sarah**	To understand key messages and findings from them	Share this more broadly
**Paul & Joanne**	Training on how to access information on past NILS projects	Identify key findings to inform their research and ensure that they do not reinvent the wheel
**Sarah, Sam, Paul & Joanne**	Training/advice on details asked for in data request submission	Complete the form effectively
**Paul/Joanne**	Training in analysis techniques for complex data structures	Conduct rigorous and robust analysis
**Paul/Joanne**	Safe researcher training	Safely analyse the data i.e. mitigate risks of disclosure
**Paul/Joanne**	To have more experience of research methods	Explore data more effectively and address more complex questions
**Fiona/Frank**	Continued training in statistical disclosure control techniques	Keep up to date with ? developments
**Paul & Joanne**	Training on NILS data	Use it effectively
**Local communities and other stakeholders**	Results/key findings from NILS analysis	Information to inform collaborative discussions and inform policies, projects and services
**Sarah and Sam**	Tools and training to use different approaches to dissemination – online – postal – meeting	Get my findings out to as wide an audience as possible
**Paul & Joanne**	Training/advice on context of NILS	Use appropriate data to answer the research questions
**Sam & Sarah**	Guidance on explaining statistics and research findings in plain language	Effectively communicate my findings to a broad audience
**Fiona and Frank**	To understand the policy context and demand	Help to show what data might help and support project delivery
**Joe**	To know how to make an application to access NILS data I have identified as relevant	Do the statistical analysis for jane

**Jane**	Advice on any constraints in respect of NILS data/output	Manage expectations of local community leaders
**Joe**	A set of tables containing hypothetical/synthetic data that is publicly available	Play with the data outside the secure environment to get a sense of that it looks like e.g., to practice linking up tables
**Joe**	SRT, Access NI accredited research status	Access the NILS data
**Jane**	To understand how social mobility could be measured and what are the associated factors determining it	Determine the extent of social mobility in my community of interest and what might be amenable to change for the better for people
**Jane**	Research support	To benchmark social mobility and inequality in communities
**Joe**	Training on NILS and how to access data	To improve understanding of NILS
**Fiona and Frank**	To deliver training and support	Help Jane and Joe to make use of NILS data
**Fiona and Frank**	To determine the experience of joe in terms of the NILS database statistical techniques, software etc.	Tailor the advice and support that I give
**Joe**	To know about the quality of certain variables through more detailed metadata	Understand why some data is missing/incomplete
**Joe**	To know what data sets can be linked to NILS	Undertake as comprehension a data analysis as possible for jane
**Fiona and frank**	Information from jane, joe and community leaders	Better understand the research context
**Fiona and frank**	Statistical disclosure control training	Clear joe's outputs quickly and efficiently
**Jane**	To understand the data	Incorporate the results into new plan
**Joe**	Safe researcher training	Access the NILS data
**Fiona and Frank**	To ensure they have the support of colleagues in other departments such as census or approvals group	Complete the project within the 8 week deadline
**Joe**	Advice on how I can make my output suitable for clearance	Complete my analysis as quickly as possible and meet project deadline

**Frank/Fiona**	Training in interdisciplinary research methods	Understand the aims of psychologists vs medics vs epidemiologists
**Joe**	Advice on the application process for NILS	Successfully apply
**Jane**	Advice on how to apply the research findings	Disseminate information effectively to policy makers
**Jane**	Advice on the interaction between community sector and health research	To ensure that the best approach to dissemination is followed
**Jane**	Basic understanding of working with NILS data	Know what is permitted and what is forbidden
**Joe**	Support/training using large datasets	Understand the data
**Fiona/frank**	Understanding of policy research	To understand how to use data to address social problems
**Jane**	Procedures for obtaining approval to use data	So they can gain access to the data they need
**Fiona/frank/joe**	Advise and support in explaining data variables and outputs	To aid proper interpretation of the study results
**Joe**	Safe researcher training	Use the NILS
**Jane**	Training on what is available through NILS – pros and cons	Choose the most suitable data source for analysis
**Jane**	Training on how to translate research findings into policy	To ensure results can be applied in the real work and translated for community audiences
**Jane**	Advice on time and practical constraints of accessing NILS	Decide if it's the best way to meet my needs.
**Joe**	Training in how to use NILS data in particular	Effectively analyse the data and follow disclosure rules
**Jane**	Training in how to write a successful application	Access the NILS data
**Fiona/frank**	Training on what RAG team looks for	Tailor and direct applications to be successful
**Joe/jane**	Advice on the administrative data environment in NI	Ensure that the best data are used to answer the research questions
**Jane/joe**	Video content/engaging method for outlining procedures and potentials of the dataset	Decide whether/if to proceed using NILS
